# Use of Robson classification to assess cesarean section rate in Brazil: the role of source of payment for childbirth

**DOI:** 10.1186/s12978-016-0228-7

**Published:** 2016-10-17

**Authors:** Marcos Nakamura-Pereira, Maria do Carmo Leal, Ana Paula Esteves-Pereira, Rosa Maria Soares Madeira Domingues, Jacqueline Alves Torres, Marcos Augusto Bastos Dias, Maria Elisabeth Moreira

**Affiliations:** 1 National Institute of Women, Children and Adolescents Health Fernandes Figueira, Oswaldo Cruz Foundation, Avenida Rui Barbosa 716 - Flamengo, CEP 22.250-020 Rio de Janeiro, RJ Brazil; 2 National School of Public Health - Oswaldo Cruz Foundation, Rua Leopoldo Bulhões, 1480, sala 809, Manguinhos, Rio de Janeiro, CEP 21041-210 Brazil; 3 National Institute of Infectology, Oswaldo Cruz Foundation, Avenida Brasil 4.365 - Manguinhos, Rio de Janeiro, RJ CEP 21.040-900 Brazil; 4 National Health Agency - Ministry of Health, Av. Augusto Severo, 84 - Glória, Rio de Janeiro, RJ 20021-040 Brazil

**Keywords:** Cesarean section, Brazil, Robson classification, Health systems

## Abstract

**Background:**

Cesarean section (CS) rates are increasing worldwide but there is some concern with this trend because of potential maternal and perinatal risks. The Robson classification is the standard method to monitor and compare CS rates. Our objective was to analyze CS rates in Brazil according to source of payment for childbirth (public or private) using the Robson classification.

**Methods:**

Data are from the 2011–2012 “Birth in Brazil” study, which used a national hospital-based sample of 23,940 women. We categorized all women into Robson groups and reported the relative size of each Robson group, the CS rate in each group and the absolute and relative contributions made by each to the overall CS rate. Differences were analyzed through chi-square and Z-test with a significance level of < 0.05.

**Results:**

The overall CS rate in Brazil was 51.9 % (42.9 % in the public and 87.9 % in the private health sector). The Robson groups with the highest impact on Brazil’s CS rate in both public and private sectors were group 2 (nulliparous, term, cephalic with induced or cesarean delivery before labor), group 5 (multiparous, term, cephalic presentation and previous cesarean section) and group 10 (cephalic preterm pregnancies), which accounted for more than 70 % of CS carried out in the country. High-risk women had significantly greater CS rates compared with low-risk women in almost all Robson groups in the public sector only.

**Conclusions:**

Public policies should be directed at reducing CS in nulliparous women, particularly by reducing the number of elective CS in these women, and encouraging vaginal birth after cesarean to reduce repeat CS in multiparous women.

**Electronic supplementary material:**

The online version of this article (doi:10.1186/s12978-016-0228-7) contains supplementary material, which is available to authorized users.

## Background

In 2015, the WHO stated that cesarean section (CS) rates higher than 10 % are not associated with reductions in maternal and newborn mortality rates and CS should ideally only be undertaken when medically necessary [1]. Nevertheless, CS rates have continued to rise worldwide and there is some concern with this trend because of the potential maternal and perinatal risks associated with CS [2–5].

Brazil is an upper-middle-income country known for its high CS rates. In 2009, for the first time, the number of CSs exceeded the number of vaginal deliveries, reaching 57 % in 2014 [6]. This difference is significantly associated with the local coverage of private health insurance, because CS rates in private hospitals (80–90 %) are considerably higher than in the public sector (35–45 %) [7–10]. It is likely that many CS performed in Brazil are for non-medical reasons [11–13].

Recently, the WHO adopted the Robson classification system as a global standard for assessing, monitoring and comparing CS rates [1]. Robson’s system classifies women into 10 groups based on five obstetric characteristics that are routinely documented: parity (nulliparous, multiparous with and without previous CS), onset of labor (spontaneous, induced or prelabor CS), gestational age (preterm or term), fetal presentation (cephalic, breech or transverse), and number of fetuses (single or multiple) [1, 14]. Compared with other CS classifications, Robson’s system offers many advantages [15]. Its categories are mutually exclusive, totally inclusive and can be applied prospectively [14, 15]. In recent years, the Robson classification has been used to analyze trends and determinants of CS rates in high- and low-income countries, such as data analysis of 21 countries included in the WHO surveys [16].

The “Birth in Brazil” study was the first national survey of obstetric and perinatal data providing a national view of labor and birth in Brazil [17]. Our objective is to assess and compare differences in CS rates according to source of payment (public or private), using Robson classification. We expect our findings to provide information to guide public policies aimed at reducing CS rate in Brazil.

## Methods

### Source of data and subjects

The “Birth in Brazil” study is a national hospital-based study of postpartum women and their newborns that was carried out from February 2011 to October 2012. This study included a complex sample of 266 hospitals with 90 postpartum women interviewed in each hospital. These hospitals were selected among those which had ≥ 500 births in 2007 (19 % of all them) and where occurred 78.6 % of all births in Brazil that year [17]. Some of facilities’ characteristics are included in Additional file 1 and further informations are presented in Azevedo Bittecourt et al. [18].

The sample was selected in three stages. In the first stage hospitals were stratified according to geographical region (North, Northeast, South, Southeast and Midwest), location (in or outside a state capital), and type of hospital health care (private, public or mixed), generating 30 stratums. Hospitals were selected with probability proportional to the number of births in each of the 30 stratums. In the second stage an inverse sampling method was used to select the number of days (minimum of seven) necessary to carry out 90 interviews of postnatal women in each hospital. In the third stage, all women who had given birth to a live newborn, regardless of weight or gestational age, or to a stillbirth with birth weight ≥ 500 g and/or gestational age ≥ 22 weeks of pregnancy in one of the sampled hospitals in the period of the data collection, were invited to participate. A calibration procedure was used to ensure that the distribution of the puerperal women interviewed was similar to that observed among the births in the population for the year 2011. Further information on the data collection [17] and the design of the sample is detailed elsewhere [19].

In the current analysis, we included all 23,894 women interviewed for the “Birth in Brazil” study.

### Robson groups and covariables

The variables necessary for applying the Robson classification are: number of fetuses (single or multiple); fetal presentation (cephalic, breech or transverse); previous obstetric record (nulliparous or multiparous, with or without uterine scar); onset of labor and delivery (spontaneous, induced or prelabor CS); and gestational age at the time of delivery.

We classified women into the 10 groups described by Robson [14] and into 12 groups using the subdivision of groups 2 and 4 to discriminate the women with induced labor from those with prelabor CS (Table 1), and eventually combined the non-cephalic groups (6, 7 and 9) to provide the analysis. We considered that women had gone into labor if they achieved at least 4 cm of cervical dilatation. Induction of labor was defined as the use of any pharmacological (oxytocin or prostaglandins) or mechanical (Foley balloon) agent in women < 4 cm dilated. The prelabor CS group included all women who had a CS and hadn’t gone into labor neither submitted to labor induction. We reported separately as group X an additional category of women not classified in a Robson group (0.03 % of all women).

**Table 1:** Characteristics of women by source of payment of birth. Birth in Brazil, 2011-2012

	Total		Public		Private		Chi square *P*-value*
	*n*	%	*n*	%	*n*	%	
Total	23,894		19,129		4,765		-
Maternal age							
< 20	4,571	19.1	4,325	22.6	246	5.2	<0.001
20–34	16,807	70.4	13,162	68.8	3,645	76.5	
> 34	2,509	10.5	1,635	8.6	874	18.3	
Skin color							
White	8,078	33.8	5,484	28.7	2,594	54.4	<0.001
Black	2,051	8.6	1,892	9.9	159	3.3	
Pardo/Mixed	13,404	56.1	11,457	59.9	1,947	40.9	
Yellow	257	1.1	202	1.1	55	1.2	
Indigenous	99	0.4	89	0.5	10,0	0.2	
Marital status							
Live with a partner	19,440	81.4	15,177	79.4	4,263	89.5	<0.001
Do not live with a partner	4,431	18.6	3,931	20.6	500	10.5	
Years of schooling							
≤ 7	6,363	26.5	6,197	32.4	166	3.5	<0.001
8 to 10	6,104	25.6	5,604	29.3	500	10.5	
11 to 14	9,310	39.0	6,790	35.5	2,520	52.9	
≥ 15	2,112	8.9	535	2.8	1,577	33.1	
Parity							
0	11,208	46.9	8,569	44.8	2,639	55.4	<0.001
1	7,015	29.4	5,405	28.3	1,610	33.8	
≥ 2	5,671	23.7	5,155	26.9	516	10.8	
Previous cesarean**							
0	7,571	59.7	6,885	65.2	686	32.2	<0.001
1	3,905	30.8	2,689	25.5	1,216	57.2	
≥ 2	1,211	9.5	986	9.3	225	10.6	
Type of pregnancy							
Single	23,610	98.8	18,936	99.0	4,674	98.1	<0.001
Multiple	284	1.2	192	1.0	92	1.9	
Induction of labor							
yes	2,729	11.4	2,561	13.4	168	3.5	<0.001
no	21,165	88.6	16,568	86.6	4,597	96.5	
Labor (spontaneous or induced)							
yes	13,458	56.3	12,618	66.0	840	17.6	<0.001
no	10,436	43.7	6,511	34.0	3,925	82.4	
Delivery							
Vaginal	11,152	46.7	10,605	55.4	547	11.5	<0.001
Forceps/Vaccum	347	1.5	317	1.7	30	0.6	
Cesarean	12,395	51.9	8,207	42.9	4,188	87.9	
High obstetric risk***							
yes	5,677	23.8	4,487	23.5	1,190	25.0	0.225
no	18,217	76.2	14,642	76.5	3,575	75.0	

* *χ*2 test

** Only women with previous cesarean

*** hypertensive disorders, eclampsia, preexisting diabetes, gestational diabetes, severe chronic diseases, infection at hospital admission for birth, placental abruption, placenta previa, intrauterine growth restriction and major newborn malformation

We defined as having a “public source of payment” those women who delivered in public health care facilities or in mixed health care facilities (private facilities financed by both public and private funds) that were not paid by a health insurance plan. “Private source of payment” included women who delivered in a mixed health care facility that was paid for by a health insurance plan and those who delivered in a private facility, regardless of whether the delivery was covered by a health insurance plan. We used the terms “public sector” and “private sector”, respectively, to refer to these definitions.

The socioeconomic, demographic and obstetric characteristics investigated were: “age” (12–19, 20–34 or ≥ 35 years); “self-reported skin color”: White, Black, Pardo/Mixed, Yellow, and Indigenous); “marital status” (living with partner or not); “duration of education” (≤7, 8–10, 11–14 and ≥ 15 years); “parity” (0, 1 or ≥ 2); “number of previous CS” (0, 1, 2, or more); “type of pregnancy” (single, multiple); “induction of labor” (yes/no); “labor (induced or spontaneous)” (yes/no); “type of delivery” (vaginal, forceps/vacuum or CS); and high obstetric risk. High obstetric risk covered the following complications: hypertensive disorders, eclampsia, preexisting diabetes, gestational diabetes, severe chronic diseases, infection at hospital admission for birth (including urinary tract infection and other sever infection such as chorioamnionitis and pneumonia), placental abruption, placenta previa, intrauterine growth restriction and major newborn malformation (including anencephaly, hidrocephaly, spina bifida, gastrosquisis and other abdominal wall defects, cardiac malformations and multiple malformations).

All data were collected from women’s and newborn medical records, except data regarding sociodemographic characteristic, such as maternal age, skin color, marital status and years of schooling, which were collected through face-to-face interviews with the mothers during their hospital stay. The gestational age was calculated using an algorithm that primarily relied upon ultrasound estimates (74 % of all women) [20].

### Statistical analysis

Differences in proportions of maternal characteristics between the public and private source of payment of birth were analyzed by chi-square statistical test with a significance level of < 0.05.

Differences in the relative size of the Robson groups by source of payment for birth (public or private) were analyzed by Z-test with Bonferroni adjustment with a significance level of < 0.05. We used the same to analyze differences in the CS rate by source of payment for birth and by obstetric risk within each Robson group.

We took into consideration the complex sampling design in all statistical analyses. The statistical program used for analysis was SPSS, version 20.0 (SPSS Inc., Chicago, IL, USA).

## Results

Only seven of the 23,894 women included in this study could not be classified into a Robson group, all of them due to uncertainty of gestational age; three of these had had a CS. The overall CS rate was 51.9 %: 42.9 % in the public and 87.9 % in the private sector. The labor induction rate was 11.4 %, and 1.2 % of women had a multiple gestation. Women covered by private payment were older and had more years of education. In this group there were also more White than Black or Pardo/Mixed women, and more women who lived with a partner, compared with those covered by public payment. There were more multiparous and fewer women with a previous CS in the public sector births. 82.4 % of women covered by private payment did not go into labor. There was no difference between the public and private sector births regarding the proportion of high-risk pregnancies (Table 1).

Table 2 shows the distribution of the women by Robson group. Almost 80 % of women were from groups 1, 2, 3 and 5, while groups 6, 7, 8 and 9 accounted for only 5 % of deliveries. The single, cephalic, preterm group (group 10) represented almost 10 % of births. Group 2 was the single largest group in the study, comprising 20 % of the whole population. Within this subset of nulliparas at term with a single cephalic infant, approximately 70 % of them were submitted to prelabor CS and nearly 30 % had labor induced. Almost 65 % of all CSs performed in Brazil were from groups 2 and 5. Groups 1, 4 and 10 contributed to 6.8 %, 8.3 % and 9.4 % of the CSs, respectively.

**Table 2:** Robson classification in Birth in Brazil study, 2011–2012

Robson group	Description of obstetric populations	Number of cesarean deliveries	Number of deliveries	Relative (%) size of group^1^	CS rate (%) in each group	Absolute contribution (%) on the overall CS rate^2^	Relative (%) contribution on the overall CS rate^3^
1	Nulliparous women, single cephalic, > = 37 weeks, in spontaneous labor	848	4,330	18.1	19.6	3.5	6.8
2	Nulliparous women, single cephalic, > = 37 weeks, induced or CS before labor	4,169	4,988	20.9	83.6	17.4	33.6
2a	Nulliparous women, single cephalic, > = 37 weeks, induced labor	618	1,437	6.0	43.0	2.6	5.0
2b	Nulliparous women, single cephalic, > = 37 weeks, CS before labor	3,551	3,551	14.9	100.0	14.9	28.6
3	Multiparous women (excluding prev. CS), single cephalic, > = 37 weeks, in spontaneous labor	264	4,775	20.0	5.5	1.1	2.1
4	Multiparous women without a previous uterine scar, with single cephalic pregnancy, > = 37 weeks, induced or CS before labor	1,028	1,685	7.1	61.0	4.3	8.3
4a	Multiparous women without a previous uterine scar, with single cephalic pregnancy, > = 37 weeks, induced labor	127	784	3.3	16.2	0.5	1.0
4b	Multiparous women without a previous uterine scar, with single cephalic pregnancy, > = 37 weeks, CS before labor	901	901	3.8	100.0	3.8	7.3
5	Previous CS, single cephalic, > = 37 weeks	3,816	4,562	19.1	83.6	16.0	30.8
6	All nullipara breeches	409	425	1.8	96.2	1.7	3.3
7	All multipara breeches (including prev. CS)	338	399	1.7	84.7	1.4	2.7
8	All multiple pregnancies (including prev. CS)	240	283	1.2	84.8	1.0	1.9
9	All abnormal lies (including prev. CS)	114	114	0.5	100.0	0.5	0.9
10	All single cephalic, <=36 weeks (including prev. CS)	1,166	2,326	9.7	50.1	4.9	9.4
X	Unable to classify	3	7	0.0	42.9	0,0	0.0
	Total	12,395	23,894	100	51.9	51.9	100

1 (Number of deliveries in the group) / (total number of deliveries)

2 (Number of cesarean deliveries in the group) / (total number of deliveries)

3 (Number of cesarean deliveries in the group) / (total number of cesarean deliveries)

Comparing the relative size of Robson groups according to source of payment, in the public sector the proportion of women in groups 1 and 3 was higher (group 1: 21.0 % vs. 6.4 %; group 3: 23.6 % vs. 5.4 %), while the private sector had a higher proportion of women in groups 2, 5 and 8 (group 2: 16.3 % vs. 39.3 %; group 5: 17.1 % vs. 27.0 %,; group 8: 1.0 % vs. 1.9 %). The proportion of women in the other groups (4, 7, 9 and 10) did not differ by source of payment of birth (Table 3).

**Table 3:** Robson group by source of payment of birth. Birth in Brazil study, 2011–2012

Robson group	Description of obstetric populations	Number of cesarean deliveries		Number of deliveries		Relative size of group^1,a^				CS rate in each group^2,b^				Relative contribution on the overall CS rate^3,c^			
		Public	Private	Public	Private	Public		Private		Public		Private		Public		Private	
						%	95 % CI	%	95 % CI	%	95 % CI	%	95 % CI	%	95 % CI	%	95 % CI
1	Nulliparous women, single cephalic, > = 37 weeks, in spontaneous labor	712	136	4,023	307	21.0	(19.5–22.2)	6.4	(5.1–7.7)	17.7	(15.1–20.6)	44.4	(35.5–53.5)	8.7	(7.5–10.1)	3.2	(2.6–4.1)
2	Nulliparous women, single cephalic, > = 37 weeks, induced or CS before labour	2,351	1,818	3,116	1,872	16.3	(15.2–17.1)	39.3	(36.2–40.7)	75.4	(71.2–79.2)	97.1	(93.3–98.8)	28.6	(26.9–30.4)	43.4	(41.2–45.7)
2ª	Nulliparous women, single cephalic, > = 37 weeks, induced labor	566	52	1,331	106	7.0	(6.1–8.0)	2.2	(1.3–3.7)	42.5	(36.8–48.4)	49.1	(29.6–69.1)	6.9	(5.7–8.3)	1.2	(0.8–1.9)
2b	Nulliparous women, single cephalic, > = 37 weeks, CS before labor	1,785	1,766	1,785	1,766	9.3	(8.5–10.3)	37.1	(34.5–39.7)	100.0	-	100.0	-	21.8	(20.3–23.3)	42.2	(39.9–44.4)
3	Multiparous women (excluding prev. CS), single cephalic, > = 37 weeks, in spontaneous labor	234	30	4,520	255	23.6	(22.3–24.6)	5.4	(4.0–6.9)	5.2	(4.1–6.5)	11.8	(7.2–18.1)	2.9	(2.3–3.5)	0.7	(0.5–1.0)
4	Multiparous without a previous uterine scar, with single cephalic pregnancy, > = 37 weeks, induced or CS before labor	758	270	1,379	306	7.2	(6.5–7.8)	6.4	(5.3–7.4)	55.0	(50.0–59.8)	88.2	(80.1–93.2)	9.2	(8.4–10.1)	6.4	(5.4–7.6)
4ª	Multiparous women without a previous uterine scar, with single cephalic pregnancy, > = 37 weeks, induced labor	118	9	739	45	3.9	(3.3–4.5)	0.9	(0.6–1.6)	16.0	(13.0–19.6)	19.5	(8.7–38.1)	1.4	(1.1–1.8)	0.2	(0.1–0.5)
4b	Multiparous women without a previous uterine scar, with single cephalic pregnancy, > = 37 weeks, CS before labor	640	261	640	261	3.3	(3.0–3.8)	5.5	(4.5–6.6)	100.0	-	100.0	-	7.8	(7.0–8.6)	6.2	(5.2–7.4)
5	Previous CS, single cephalic, > = 37 weeks	2,556	1,260	3,276	1,286	17.1	(16.0–18.0)	27.0	(24.8–28.1)	78.0	(75.4–80.4)	98.0	(96.5–98.9)	31.1	(29.7–32.7)	30.1	(28.5–31.8)
6	All nullipara breeches	271	138	286	139	1.5	(1.3–1.7)	2.9	(2.0–4.0)	94.4	(91.2–96.9)	99.3	(96.5–99.9)	3.3	(2.8–3.8)	3.3	(2.3–4.6)
7	All multipara breeches (including prev. CS)	276	62	336	63	1.8	(1.5–2.1)	1.3	(1.0–1.7)	82.1	(71.9–89.1)	98.4	(93.0–99.6)	3.4	(2.9–3.9)	1.5	(1.2–1.9)
8	All multiple pregnancies (including prev. CS)	153	87	191	92	1.0	(0.8–1.2)	1.9	(1.5–2.6)	79.7	(72.1–85.6)	94.6	(72.7–99.1)	1.9	(1.5–2.3)	2.1	(1.5–3.0)
9	All abnormal lies (including prev. CS)	91	23	91	23	0.5	(0.4–0.6)	0.5	(0.3–0.8)	100.0	-	100.0	-	1.1	(0.9–1.4)	0.5	(0.3–0.9)
10	All single cephalic, <=36 weeks (including prev. CS)	803	363	1,904	422	10.0	(8.7–11.2)	8.9	(7.1–10.6)	42.2	(38.7–45.7)	86.0	(78.3–91.1)	9.8	(8.3–11.5)	8.7	(6.9–10.9)
X	Unable to classify	2	1	6	1	0.03	(0.01–0.07)	0.02	(0.002–0.01)	40.2	(7.8–84.2)	100.0	-	0.0	-	0.0	-
	Total deliveries	8,207	4,188	19,128	4,766	100.0	-	100.0	-	41.5	-	87.9	-	100.0		100.0	

^1^ (Number of deliveries in the group) / (total number of deliveries)

^2^ (Number of cesarean deliveries) / (number of deliveries in the same Robson group)

^3^ (Number of cesarean deliveries in the group) / (total number of cesarean deliveries)

^a^ Public vs. Private proportions differed significantly from each other at the .05 level by means of z-test with Bonferroni adjustment for all Robson group, except for groups 4, 7, 9, 10 and X

^b^ Public vs. Private proportions differed significantly from each other at the .05 level by means of z-test with Bonferroni adjustment for all Robson group, except for groups 2a, 4a and 8

^c^ Public vs. Private proportions differed significantly from each other at the .05 level by means of z-test with Bonferroni adjustment for all Robson group, except for groups 4b, 5, 6, 8 and 10

The analysis of CS rates by group showed that within Robson group 1 (nulliparous, cephalic, term, spontaneous labor), the CS rate was more than two-fold higher in the private than the public sector (44.4 % in private and 17.7 % in public), and the same occurred within group 10 (all single cephalic, ≤ 36 weeks; 86.0 % in private and 42.2 % in public). The CS rate in groups 2a and 4a was also not different between the public and private sector. However, there was a difference when all women from groups 2 and 4 were considered (group 2: 75.4 % in public and 97.1 % in private; group 4: 55.0 % in public and 88.2 % in private). Analyzing the relative contribution of the groups to the CS rate showed that there were statistical differences for all groups of cephalic term without previous CS (groups 1 to 4), while groups 5 and 10 contributed a similar percentage in the public and private sectors (Table 3).

**Fig. 1:**
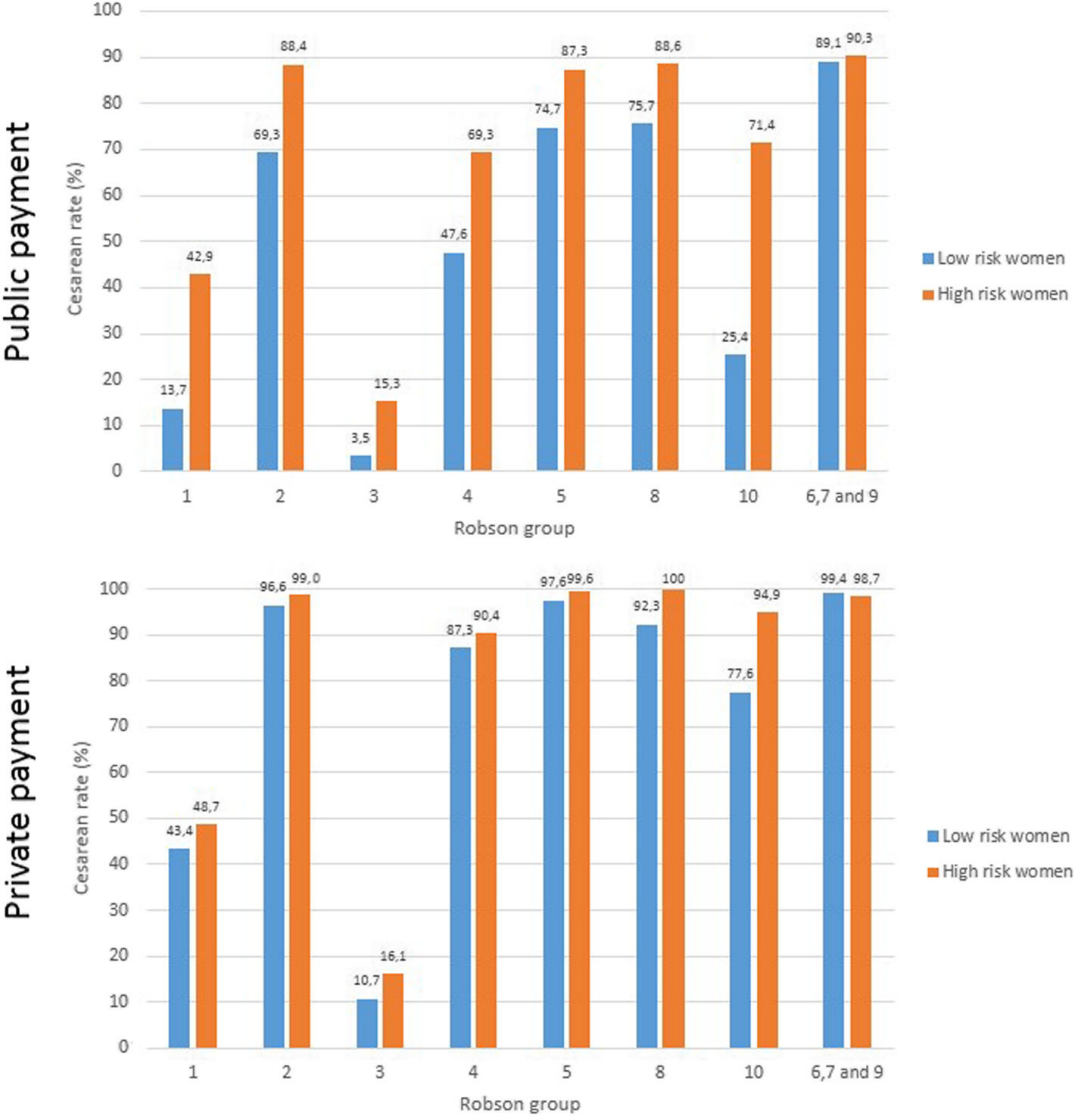
Cesarean rates into Robson groups according to obstetric risk (low-risk women^1^ and high-risk^2^ women in public and private sectors. 1 women without any of high risk characteristics. 2 hypertensive disorders, eclampsia, preexisting diabetes, gestational diabetes, severe chronic diseases, infection at hospital admission for birth, placental abruption, placenta previa, intrauterine growth restriction and major newborn malformation

In the public sector, CS rates were statistically greater in women at high obstetric risk (67.7 %) compared with women of low obstetric risk (35.3 %). This was true for most Robson categories, except for the non-cephalic groups (6, 7 and 9 combined). In the private sector, there were no statistically significant differences in CS rates when high-obstetric-risk women (92.8 %) were compared with low-obstetric-risk women (86.3 %), except for categorie 10 (Table 4 and Fig. 1).

**Table 4:** Caesarean section rates (%) per Robson group in high and low risk women according to source of payment. Birth in Brazil study, 2011–2012

Robson group	Public								Private							
	Low risk women^1^				High risk women^2^				Low risk women^1^				High risk women^2^			
	All	CS	%	95 % CI^a^	All	CS	%	95 % CI^a^	All	CS	%	95 % CI^b^	All	CS	%	95 % CI^b^
All groups	14,640	5,168	35.3	(33.0–37.6)	4,484	3,036	67.7	(64.9–70.4)	3,573	3,082	86.3	(81.2–90.1)	1,190	1,104	92.8	(89.5–95.0)
1	3,475	477	13.7	(11.2–16.6)	550	236	42.9	(36.8–49.2)	253	110	43.4	(33.8–53.4)	54	26	48.7	(29.9–67.8)
2	2,114	1,465	69.3	(63.9–74.2)	1,002	885	88.4	(85.2–91.0)	1,425	1,376	96.6	(91.4–98.7)	444	441	99.0	(96.9–99.7)
3	3,886	137	3.5	(2.6–4.7)	636	97	15.3	(11.2–20.4)	214	23	10.7	(6.5–17.2)	41	7	16.1	(7.6–30.9)
4	911	434	47.6	(42.1–53.2)	467	324	69.3	(62.9–75.1)	220	192	87.3	(77.7–93.1)	85	77	90.4	(82.2–95.0)
5	2,410	1,799	74.7	(71.3–77.8)	866	756	87.3	(84.3–89.7)	1,002	978	97.6	(95.7–98.7)	284	283	99.6	(97.5–100.0)
8	125	95	75.7	(66.4–83.1)	67	59	88.6	(79.6–93.9)	67	62	92.3	(63.0–98.8)	25	25	100.0	-
10	1,208	306	25.4	(21.8–29.2)	694	497	71.4	(65.4–76.8)	222	172	77.6	(67.5–85.3)	201	190	94.9	(88.3–97.9)
6, 7 and 9	511	455	89.1	(81.4–93.9)	202	182	90.3	(83.0–94.6)	170	169	99.4	(97.4–99.9)	56	55	98.7	(91.4–99.8)

^1^women without any of high risk characteristics

^2^hypertensive disorders, eclampsia, preexisting diabetes, gestational diabetes, severe chronic diseases, infection at hospital admission for birth, placental abruption, placenta previa, intrauterine growth restriction and major newborn malformation

^a^Low risk women vs. high risk women proportions of CS differed significantly from each other at the .05 level by means of z-test with Bonferroni adjustment for all Robson groups, except for groups 6, 7 and 9 combined

^b^Low risk women vs. high risk women proportions of CS DID NOT differ significantly from each other at the .05 level by means of z-test with Bonferroni adjustment for all Robson group, except for group 10

## Discussion

### Main findings

The CS rate in Brazil was more than two-fold higher in women covered by private health care than in women who delivered in the public sector. The groups with the greatest impact on Brazil’s CS rate in both public and private sectors were group 2 (nulliparous, term, cephalic with induced or cesarean delivery before labor), group 5 (multiparous, term, cephalic presentation and previous cesarean section) and group 10 (cephalic preterm pregnancies), which accounted for more than 70 % of CS carried out in the country.

The prevalence of obstetric risk was not different despite the discrepancies in sociodemographic characteristics of women from the public and private sectors. High-risk women had significantly higher CS rates when compared with low-risk women in almost all Robson groups only in the public sector, but not in the private sector, which suggest a liberally overuse of CS in women with private health care.

### Strengths and limitations

This study is important for many reasons. First, it was based on a national survey that covers all Brazilian states and was representative of 2,337,475 births (80 %) occurring in 2011 [19]. To our knowledge, it is the third study that used the Robson classification to assess CS rates at a national level and the second to use primary data [21, 22]. We collected all essential information included in the Robson classification, and only a few women could not be classified into one of the Robson groups. This minimized the problem of using routine data, which are not always accurate. Second, we estimated gestational age using an algorithm, based primarily on obstetric ultrasound, which confers certain advantages over last menstrual period, as the latter tends to overestimate the rate of preterm birth in the Brazilian population [20]. Finally, we also used a clear definition to classify women who went into labor, which is commonly lacking in previous reports [23].

Because of the sample design, our results can only be extrapolated to the 80 % of the population who give birth in hospitals with more than 500 deliveries per year, and not to the entire Brazilian population. In addition, this study had limited power to compare differences between the public and private sectors for categories of Robson groups of very low frequency, such as categories 6, 7, 8, 9 and induction groups in the private sector (2a and 4a). Another limitation of the study is the potential misclassification of some women who belonged in Groups 1 and 3 and were erroneously classified as Groups 2 and 4 because of the definition used for labor induction. It is possible that some nulliparous and multiparous women admitted with spontaneous onset of labor (Groups 1 and 3) received oxytocin during the latent phase, before reaching 4 cm dilation, for augmentation of labor. However, this probably will not affect the main findings of the study, considering the underuse of labor induction in this study.

### Interpretation

CS rates continue to increase around the world without a clear understanding of the main drivers and consequences. The CS rate found in the “Birth in Brazil” study (51.9 %) is among the highest in the world along with China (52.5 %), Cyprus (52.2 %), the Dominican Republic (56.4 %) and Egypt (51.8 %) [24, 25]. There is evidence that it continues to grow [6].

Our results showed that women who delivered in the private sector were more frequently white, older and with higher education, conditions associated with CS in previous studies [26, 27]. Although there were more multiparous women, and fewer twin pregnancies and previous CS in women in the public sector, it is unlikely that these factors alone can explain the difference in CS rates. The low use of labor induction in the private sector (only 3.5 %) was also remarkable, reinforcing the preference for CS before labor as a form of immediate delivery. Even in the public sector, the rate of induced deliveries was lower than in countries with low CS rates, such as France and the Netherlands [21, 22], and also lower than previously reported in Latin America [28].

The current analysis of CS by Robson classification revealed, as with other studies, that the nulliparous group, term, cephalic presentation is one that contributes most to the total rate of CS [21, 29, 30]. Analyzing nine institutions, Brennan et al. [29] showed that 98 % of institutional variation in the CS rate may be attributed to this group, which contributed to over 30 % of CSs performed in France and the Netherlands [21, 22]. The same authors also pointed out that the proportion of this group in the population was similar between institutions, reinforcing the hypothesis that there are variations in the CS rate in this group that affect the overall rate. In our study, the proportion of groups 1 and 2 combined was 39 %, similar to that found in Latin America (36.4 %) [31], France (38.2 %) [21], Canada (39.7 %) [30] and the Netherlands (39.9 %) [22]. However, in Brazil, we found that the group of CSs before labor (group 2b) impacted more on the contribution of term nulliparous women (14.9 %). In European countries, the proportion of this group (2b) is around 1 % of the obstetric population [21, 22], but even in the Brazilian public sector, this group included 9 % of women in our study. As the number of nulliparous women is almost the same, the proportion of group 1 (18.9 %) was below what is commonly found in other studies that have reported it at above 25 % of the obstetric population [21, 22, 29, 31]. When we analyzed the women with private payment, this percentage was even lower (6.4 %), despite the higher proportion of nulliparous, term, cephalic women in the private (45.7 %) than the public (36.1 %) sector.

The group that singly most contributed to CS in Brazil was multiparous, term with previous CS (group 5). Recently, a WHO analysis found that CS rate and the absolute contribution of group 5 has increased in recent years [16]. These data show the domino effect of CS use: rising CS rates, especially in nulliparous women, increase the number of women with previous CS, who are more likely to undergo a repeat CS [16]. As a result of the history of high CS rates in Brazil, group 5 constitutes almost 20 % of Brazil’s population; combined with the high rate of repeat CS, this makes it responsible for almost a third of CS carried out in the country both in the public and private sectors. Our data are consistent with the WHO Global Survey of Latin America [31], where group 5 accounted for 26.7 % of CS. The CS rate for this group, although not different from that found in countries with very high and high human development index in the WHO surveys (from 78.1 to 79.4 %) [19], is considerably higher than that found in France (61 %) [21] and the Netherlands (47 %) [22]. While the success of vaginal birth after cesarean (VBAC) reaches 70 % in many studies [32], an incentive to this practice would be essential to reduce CSs in Brazil. In addition, repeat CSs increase the chance of placenta accreta and placenta previa, which can result in increased risk in subsequent pregnancies [32, 33].

The multiparous groups without CS (groups 3 and 4) contributed to just over 10 % of CSs. Noteworthy is the high CS rate in group 4 (61 %), even in the public sector (55 %), which is related to the number of women undergoing CS before delivery (group 4b) that is greater than those undergoing induction (group 4a). While in Brazil group 4b corresponds to 3.2 % of women, in other countries it does not exceed 1 % [21, 22, 30]. These numbers may again reflect the preference of CS to induction of labor in high-risk pregnancies, but also the use of CS for concomitant tubal ligation, as mentioned in other reviews [8, 11, 34].

The third group that contributed most to the CS rate in both sectors was the preterm birth group, contributing to nearly 10 % of CSs performed in Brazil. This number is slightly higher than that found in countries with low rates of prematurity. In the Netherlands, group 10 corresponds to 7.1 % of CS [22], while in France the percentage is 8.3 % [21]. In Brazil, both the group size (9.7 %) and its CS rate (50.1 %) affected the CS overall rate.

Finally, the groups of non-cephalic presentations (groups 6, 7 and 9) and twins (group 8) together contributed only 8.9 % of CSs. This number is lower than the WHO Survey of Latin America (14 %) [31] and considerably lower than that observed in France (20.5 %) [21] and the Netherlands (27.2 %) [22]. Even excluding twins, whose prevalence in these countries is greater, and considering only non-cephalic presentations, the gap remains large (Brazil: 7 %; France: 16.5 %; Netherlands: 22.5 %).

In Brazil, there was a clear difference in both the distribution of women and CS rates into Robson groups according to the source of payment. The two largest relative size groups in the public sector (groups 3 and 1) had little importance in the private sector. Additionally, there was a clear concentration of nulliparous women in group 2b and multiparous women in group 5, which represented > 70 % of CSs in the private sector, where > 80 % of women did not go into labor, reinforcing the saying “once a cesarean, always a cesarean.”

Analyzing the increase in the number of CSs in the period between the two WHO surveys, Vogel et al. [16] concluded that the threshold for medically indicated CS has become lower over time, or the use of elective CS has risen, or both occurred together. This appears to be what has occurred in Brazil over recent decades. While the CS rate is higher than those found in other countries in groups with low probability of CS (term nulliparous and multiparous with spontaneous labor and multiparous with induced labor), the widespread use of elective CS in nulliparous and multiparous women, regardless of obstetric risk, even in the public sector, was also observed. Indeed, 84.2 % of all CS in Brazil are performed before the active phase of labor (data not shown).

In the private sector, it is very likely that CS was not related to the presence of obstetric risk, since CS rates according to the risk of pregnancy were different only in group 10. Furthermore, CS rates were also extremely high in low-risk women. Despite women with private funding having a greater preference for CS (36.1 % of nulliparous and 58.8 % of multiparous in early pregnancy) [8], this fact alone does not explain such high rates of CS.

The high rates of elective CS in Brazil, especially in the private sector, are of concern, because they may bring unnecessary harm to women’s and babies’ health if performed without indication [5], including increased maternal [3] and neonatal morbidity, especially when performed before 39 weeks [35]. Our data revealed a great difference in CS rates in the low-risk preterm group according to source of payment (25.4 % public and 71.4 % private), which raises questions about whether this practice may be leading to iatrogenic prematurity.

## Conclusions

This is an analysis of CS rates in Brazil by Robson classification using data from the entire country. The Robson classification identifies the contributors to the CS rate, but does not provide insight into the reasons or explanation for the observed differences [23]. However, this classification helps to identify the target groups that may benefit from implementations or interventions, and guide public policies and investments for reducing CS rates in Brazil.

Public policies should be directed at reducing CSs in nulliparous women, particularly by reducing the number of elective CSs in these women. The extended use of labor induction and its appropriate management rather than CS before labor would be an important measure for reducing CS rates. Encouraging VBAC and reducing repeat CSs are equally important, since > 70 % of CSs carried out in the country were in these groups. These policies should also be directed at the private sector, where one third of all CSs are performed in Brazil and where this surgery indication seems not be driven by medical reasons.

## Additional files

Additional file 1: Characteristics of facilities according to type of financing. Birth in Brazil, 2011-2012. (DOC 53 kb)
Additional file 2: Translated article. (DOCX 101 kb)
Additional file 3: Reviewer reports. (PDF 293 kb)
