# Supplementary material for: Use of Robson classification to assess cesarean section rate in Brazil: the role of source of payment for childbirth
Source: Reprod Health. 2016 Oct 17;13(Suppl 3):128. doi: 10.1186/s12978-016-0228-7 (PMC5073850; doi:10.1186/s12978-016-0228-7)
Supplement: Additional file 1: — Characteristics of facilities according to type of financing. Birth in Brazil, 2011-2012. (DOC 53 kb) [file 12978_2016_228_MOESM1_ESM.doc]

Additional Table 1 - Characteristics of facilities according to type of financing. Birth in Brazil, 2011-2012

|  | Public or Mixed* | | Private | |
| --- | --- | --- | --- | --- |
|  | n | % | n | % |
| **Total** | **218** | **100** | **48** | **100** |
| **Births per year** |  |  |  |  |
| ≥ 3000 | 38 | 17,5 | 4 | 8,7 |
| 1000 to 2999 | 112 | 51,3 | 23 | 48,8 |
| ≤ 999 | 68 | 31,2 | 21 | 42,5 |
| **ICU (maternal or neonatal)** |  |  |  |  |
| yes | 124 | 56,9 | 42 | 87,5 |
| no | 94 | 43,1 | 6 | 12,5 |
| **Neonatal ICU** |  |  |  |  |
| yes | 86 | 39,2 | 38 | 79,2 |
| no | 132 | 60,8 | 10 | 20,8 |
| **Maternal ICU** |  |  |  |  |
| yes | 113 | 51.8 | 36 | 74.9 |
| no | 105 | 48.2 | 12 | 25.1 |
| **Mechanical ventilator (adult)** |  |  |  |  |
| Avaiable | 181 | 83,0 | 47 | 98,8 |
| Not avaiable | 37 | 17,0 | 1 | 1,2 |
| **Material for neonatal ventilation** |  |  |  |  |
| Avaiable | 208 | 95.6 | 47 | 98.0 |
| Not avaiable | 10 | 4.4 | 1 | 2.0 |
| **Transfusion unit or Blood bank** |  |  |  |  |
| yes | 158 | 72.3 | 32 | 66.8 |
| no | 60 | 27.7 | 16 | 33.2 |
| **Laboratory tests** |  |  |  |  |
| Avaiable | 191 | 87.8 | 41 | 86.6 |
| Not avaiable | 27 | 12.2 | 7 | 13.4 |
|  |  |  |  |  |
| * Mixed hospitals represents 45.6% of all facilities and 88.3% of births in these hospital had public source of payment | | | | |
