# Supplementary material for: Use of Robson classification to assess cesarean section rate in Brazil: the role of source of payment for childbirth
Source: Reprod Health. 2016 Oct 17;13(Suppl 3):128. doi: 10.1186/s12978-016-0228-7 (PMC5073850; doi:10.1186/s12978-016-0228-7)
Supplement: Additional file 2: — Translated article. (DOCX 101 kb) [file 12978_2016_228_MOESM2_ESM.docx]

**O uso da classificação de Robson para avaliar a taxas de cesariana no Brasil: o papel da fonte de pagamento para o parto**

**Autores:** Marcos Nakamura-Pereira^1,2^, Maria do Carmo Leal^2^, Ana Paula Esteves-Pereira^2^, Rosa Maria Soares Madeira Domingues^3^, Jacqueline Alves Torres^4^, Marcos Augusto Bastos Dias^1^ e Maria Elisabeth Moreira^1^

**Marcos Nakamura-Pereira (autor para correspondência)**

1 Instituto Nacional de Saúde da Mulher, da Criança e do Adolescente Fernandes Figueira, Fundação Oswaldo Cruz, Rio de Janeiro, Brasil. Avenida Rui Barbosa 716 - Flamengo, Rio de Janeiro, RJ. CEP 22250-020. e-mails: marcosnakamura@globo.com, marcosad@centroin.com.br, [bebethiff@gmail.com](mailto:bebethiff@gmail.com). Telefone: +55 (21) 2554-1700.

2 Escola Nacional de Saúde Pública - Fundação Oswaldo Cruz, Rio de Janeiro, Brasil. Rua Leopoldo Bulhões, 1480, sala 809, Manguinhos - Rio de Janeiro – CEP 21041-210. e-mails: [ducaleal@gmail.com](mailto:ducaleal@gmail.com); [ana.pep@gmail.com](mailto:ana.pep@gmail.com). Telefone: +55 (21) 2598-2620

3 Instituto Nacional de Infectologia, Fundação Oswaldo Cruz, Rio de Janeiro, Brasil. Avenida Brasil 4365 - Manguinhos, Rio de Janeiro, RJ. CEP 21040-900 e-mail: [rosamsmd@gmail.com](mailto:rosamsmd@gmail.com). Telefone: +55 (21) 3865-9595

4 Agência Nacional de Saúde – Ministério da Saúde. Av. Augusto Severo, 84 - Glória, Rio de Janeiro - RJ, 20021-040, Brasil. e-mail:jaqueline.torres@ans.gov.br. Telefone: +55 21 2105-0000.

**RESUMO**

*Introdução*: As taxas de cesárea (CS) estão aumentando em todo o mundo, mas existe alguma preocupação com essa tendência, por causa do seu potencial risco materno e perinatal. A classificação Robson é o método padrão para monitorar e comparar as taxas de cesárea. Nosso objetivo foi analisar as taxas de cesárea no Brasil, segundo a fonte de pagamento para o parto (pública ou privada) usando a classificação de Robson.

*Métodos*: Os dados são do estudo "Nascer no Brasil", realizado em 2011-2012, que utilizou uma amostra de base hospitalar nacional de 23.940 mulheres. Categorizamos todas as mulheres em grupos Robson e descrevemos o tamanho relativo de cada grupo de Robson, a taxa de CS em cada grupo e as contribuições absolutas e relativas de cada um para a taxa global de CS. As diferenças foram analisadas ​​por meio do teste qui-quadrado e Z-teste com nível de significância <0,05.

*Resultados*: A taxa global de CS no Brasil foi de 51,9% (42,9% no setor público e 87,9% no setor privado de saúde). Os grupos Robson com maior impacto na taxa de CS do Brasil em ambos os setores público e privado foram o grupo 2 (nulíparas, termo, apresentação cefálica com parto induzido ou cesariana anteparto), grupo 5 (multíparas, termo, apresentação cefálica e cesariana anterior) e grupo 10 (gravidezes prematuras cefálicas), que responderam por mais de 70% das CS realizadas no país. Mulheres de alto risco tiveram significativamente maiores taxas de cesárea em comparação com as mulheres de baixo risco em quase todos os grupos de Robson somente no setor público.

*Conclusões*: As políticas públicas devem ser dirigidas a reduzir as CS em mulheres nulíparas, especialmente através da redução do número de CS eletivas nestas mulheres, e incentivar parto vaginal após cesariana para reduzir a CS de repetição em mulheres multíparas.

**Palavras-chave:** cesariana, Brasil, classificação Robson, sistemas de saúde.

**INTRODUÇÃO**

Em 2015, a OMS declarou que taxas de cesariana acima de 10% não estão associadas a reduções da mortalidade materna e neonatal e as cesarianas devem, idealmente, ser realizadas quando indicadas por motivos médicos [1]. No entanto, as taxas de cesárea continuam a aumentar em todo o mundo e existe alguma preocupação com esta tendência devido aos potenciais riscos maternos e perinatais associados a este procedimento [2-5].

O Brasil é um país de renda média superior conhecido por suas altas taxas de cesárea. Em 2009, pela primeira vez, o número de cesarianas excedeu o número de partos vaginais, atingindo 57% em 2014 [6]. Esta diferença é significativamente associada com a cobertura local de plano de saúde privado, porque as taxas de cesárea em hospitais privados (80-90%) são consideravelmente mais elevadas do que no setor público (35-45%) [7-10]. É provável que muitas cesáreas realizadas no Brasil sejam por razões não médicas [11-13].

Recentemente, a OMS adotou o sistema de classificação Robson como um padrão global para avaliar, controlar e comparar as taxas de cesariana [1]. A classificação de Robson distribui as mulheres em 10 grupos com base em cinco características obstétricas que são rotineiramente documentadas: paridade (nulíparas, multíparas, com e sem cesárea anterior), o início do trabalho de parto (espontâneo, induzido ou cesárea anteparto), idade gestacional (pré-termo ou termo), apresentação fetal (cefálica, pélvica ou transversa) e o número de fetos (única ou múltipla) [1,14]. Em comparação com outras classificações de cesariana, o sistema de Robson oferece muitas vantagens [15]. Suas categorias são mutuamente exclusivas, totalmente inclusivas e podem ser aplicadas prospectivamente [14,15]. Nos últimos anos, a classificação Robson vem sendo utilizada para analisar tendências e determinantes das taxas de CS em países de alta e de baixa renda, como a análise de dados de 21 países incluídos nas pesquisas da OMS [16].

O estudo "Nascer no Brasil" foi a primeira pesquisa nacional de dados obstétricos e perinatais fornecendo uma visão nacional do parto e nascimento no Brasil [17]. Nosso objetivo é avaliar e comparar diferenças nas taxas de cesariana de acordo com a fonte de pagamento (público ou privado), utilizando a classificação Robson. Esperamos que nossos resultados possam fornecer informações para orientar as políticas públicas destinadas a reduzir a taxa de cesariana no Brasil.

**MÉTODOS**

**Fontes de dados e sujeitos**

O estudo "Nascer no Brasil" é um estudo nacional de base hospitalar com puérperas e seus recém-nascidos, que foi realizado no período de fevereiro de 2011 a outubro de 2012. Este estudo incluiu uma amostra complexa de 266 hospitais com 90 puérperas entrevistadas em cada hospital. Estes hospitais foram selecionados entre aqueles que tiveram ≥ 500 nascimentos em 2007 (19% de todos eles) e onde ocorreram 78,6% de todos os nascimentos no Brasil naquele ano [17]. Algumas das características dos hospiatais estão incluídas no *Arquivo adicional 1* e maiores informações estão apresentadas em Azevedo Bittecourt et al. [18].

A amostra foi selecionada em três etapas. No primeiro estágio, os hospitais foram estratificados de acordo com a região geográfica (Norte, Nordeste, Sul, Sudeste e Centro-Oeste), localização (dentro ou fora de uma capital do estado), e tipo de hospital (privado, público ou misto), gerando 30 estratos. Os hospitais foram selecionados de acordo com a probabilidade proporcional ao número de nascimentos em cada um dos 30 estratos. Na segunda etapa, um método de amostragem inversa foi usado para selecionar o número de dias (mínimo de sete) necessários para realizar 90 entrevistas de mulheres no puerpério em cada hospital. Na terceira etapa, todas as mulheres que tinham dado à luz a um recém-nascido vivo, independentemente do peso ou idade gestacional, ou a um natimorto com peso de nascimento ≥ 500 g e/ou idade gestacional ≥ 22 semanas, em um dos hospitais incluídos na amostra o período da coleta de dados, foram convidados a participar. Um procedimento de calibração foi usado para assegurar que a distribuição das puérperas entrevistadas fosse semelhante à observada entre os nascimentos na população para o ano 2011. Mais informações sobre a coleta de dados [17] e a concepção da amostra [19] estão detalhadas em outros artigos.

Na atual análise, foram incluídas todas as 23,894 mulheres entrevistadas para o estudo "Nascer no Brasil".

**Grupos de Robson e covariáveis**

As variáveis ​​necessárias para a aplicação da classificação de Robson são: número de fetos (único ou múltiplos); apresentação fetal (cefálica, pélvica ou córmica); história obstétrica (nulíparas ou multíparas, com ou sem cicatriz uterina); início do trabalho de parto (espontâneo, induzido ou cesárea anteparto); e idade gestacional no momento do nascimento.

Classificamos as mulheres nos 10 grupos descritos por Robson [14] e em 12 grupos que utilizam a subdivisão dos grupos 2 e 4 para discriminar as mulheres com trabalho de parto induzido daquelas com cesárea anteparto (Tabela 1), e, eventualmente, combinamos os grupos não-cefálicos (6, 7 e 9) para realizar a análise. Considerou-se que as mulheres tinham apresentado trabalho de parto, caso alcançassem pelo menos 4 cm de dilatação cervical. Indução do parto foi definida como o uso de qualquer método farmacológico (ocitocina ou prostaglandinas) ou mecânico (balão de Foley) em mulheres con < 4 cm de dilatação. O grupo de cesárea anteparto incluiu todas as mulheres que tiveram uma cesariana e não apresentaram trabalho de parto, nem foram submetidas à indução do parto. Descrevemos como grupo X as mulheres que não foram classificadas em nenhum dos grupos de Robson (0,03 % de todas as mulheres).

Definiu-se como tendo "fonte pública de pagamento" aquelas mulheres que deram à luz em unidades de saúde públicas ou em centros de saúde mistos (instalações privadas financiadas por fundos públicos e privados) que não foram pagos por um plano ou seguro de saúde. "Fonte privada de pagamento" incluiu mulheres que deram à luz em uma unidade de saúde mista que foi paga por um plano de saúde e aquelas que tiveram o parto em uma instituição privada, independentemente se o parto foi coberto por plano de saúde ou não. Nós usamos os termos "setor público" e "setor privado", respectivamente, para se referir a essas definições.

As características socioeconômicas, demográficas e obstétricas investigados foram: "idade" (12-19, 20-34 ou ≥ 35 anos); "cor da pele autorreferida": branca, preta, parda/morena, amarela e indígena); "estado marital" (vivendo com o parceiro ou não); "escolaridade" (≤ 7, 8-10, 11-14 e ≥ 15 anos); "paridade" (0, 1 ou 2 ≥); "número de cesarianas anteriores" (0, 1, 2 ou mais); "tipo de gravidez" (única, múltipla); "indução do parto" (sim / não); "início do trabalho de parto (induzido ou espontâneo)" (sim / não); "tipo de parto" (vaginal, fórceps / vácuo ou cesariana); e alto risco obstétrico (sim / não). O alto risco obstétrico incluiu as seguintes complicações: síndormes hipertensivas, eclâmpsia, diabetes pré-gestacional, diabetes gestacional, doenças crónicas graves, infecção no momento da internação para o parto (incluindo infecção do trato urinário e outra infecção grave, como corioamnionite e pneumonia), descolamento prematuro da placenta, placenta prévia, crescimento intrauterino restrito e malformações fetais (incluindo anencefalia, hidrocefalia, espinha bífida, gastrosquise e outros defeitos da parede abdominal, malformações cardíacas e malformações múltiplas).

Todos os dados foram coletados dos prontuários de mulheres e recém-nascidos, exceto os dados sobre características sociodemográficas, como idade materna, cor da pele, estado marital e escolaridade, que foram coletados através de entrevistas face-a-face com as mães durante a internação. A idade gestacional foi calculada usando um algoritmo que se baseou principalmente em estimativas de ultrassom (74% de todas as mulheres ) [20].

**Análise estatística**

Diferenças de proporções das características maternas entre as fontes de pagamento público e privado foram analisadas ​​pelo teste estatístico qui-quadrado com nível de significância de < 0,05.

Diferenças no tamanho relativo dos grupos de Robson por fonte de pagamento (público ou privado) foram analisadas ​​por Z-teste com ajuste de Bonferroni e nível de significância < 0,05. Usamos o mesmo teste para analisar as diferenças na taxa de cesariana por fonte de pagamento para o parto e por risco obstétrico dentro de cada grupo de Robson.

Levou-se em consideração o desenho amostral complexo em todas as análises estatísticas. O programa estatístico utilizado para análise foi o SPSS, versão 20.0 (SPSS Inc., Chicago, IL , EUA).

**Considerações éticas**

Esta pesquisa foi conduzida conforme o Conselho Nacional de Saúde, que fornece orientações e normas de pesquisa com seres humanos, sob o protocolo de pesquisa CEP / ENSP - n ° 92/10. Todo cuidado foi tomado para assegurar a privacidade e confidencialidade das informações. Obtivemos a aprovação dos comitês de ética em pesquisa de cada um dos 266 hospitais participantes. Todos os diretores dos hospitais e mulheres incluídas no estudo assinaram consentimento informado.

**RESULTADOS**

Apenas sete das 23.894 mulheres incluídas neste estudo não puderam ser classificadas em um dos grupos de Robson, todas elas devido à incerteza da idade gestacional; três delas foram submetidas à cesariana. A taxa global de cesárea foi de 51,9%: 42,9% no setor público e 87,9% no setor privado. A taxa de indução do parto foi de 11,4% e 1,2% das mulheres tiveram gestação múltipla. Mulheres com cobertura de plano de saúde privado eram mais velhas e tinham mais anos de escolaridade. Neste grupo também houve mais brancas do que pretas ou pardas, e mais mulheres que viviam com parceiro, em comparação com aquelas cuja fonte de pagamento do parto foi pública. Havia mais mulheres multíparas e menos com a cesárea anterior no setor público. 82,4% das mulheres com fonte de pagamento do parto privado não entraram em trabalho de parto. Não houve diferença entre os nascimentos dos setores público e privado em relação à proporção de gravidezes de alto risco (Tabela 1).

A Tabela 2 mostra a distribuição das mulheres por grupo de Robson. Quase 80% das mulheres eram dos grupos 1, 2, 3 e 5, enquanto os grupos 6, 7, 8 e 9 representaram apenas 5% dos partos. O grupo único, cefálico, pré-termo (grupo 10) abrangeu quase 10% dos nascimentos. O grupo 2 foi o maior grupo neste estudo, compreendendo 20% de toda a população. Dentro deste subconjunto de nulíparas a termo com gestação única cefálica, cerca de 70% delas foram submetidas à cesárea anteparto e quase 30% tiveram o parto induzido. Quase 65% de todas as cesarianas realizadas no Brasil foram dos grupos 2 e 5. Os grupos 1, 4 e 10 contribuíram para 6,8%, 8,3% e 9,4 % das cesarianas, respectivamente.

Comparando o tamanho relativo dos grupos Robson de acordo com a fonte de pagamento, no setor público, a proporção de mulheres nos grupos 1 e 3 foi maior (grupo 1: 21,0% vs. 6,4%; grupo 3: 23,6 % vs. 5,4%), enquanto o setor privado teve uma proporção maior de mulheres em grupos de 2 , 5 e 8 (grupo 2: 16,3% vs. 39,3%; grupo 5: 17,1% vs. 27,0%; grupo 8: 1,0 % vs. 1,9%). A proporção de mulheres nos outros grupos (4, 7, 9 e 10) não diferiu por fonte de pagamento para o parto (Tabela 3).

A análise das taxas de cesárea por grupo mostrou que dentro do grupo 1 de Robson (nulíparas, cefálica, termo, trabalho de parto espontâneo), a taxa de cesárea foi mais de duas vezes maior no setor privado do que no setor público (44,4% no privado e 17,7% no público), o mesmo ocorrendo dentro do grupo 10 (todos os únicos cefálicos, ≤ 36 semanas; 86,0% no privado e 42,2% no público). As taxas de cesárea nos grupos 2a e 4a não foram diferentes entre os setores público e privado. No entanto, houve diferença quando todas as mulheres de grupos 2 e 4 foram consideradas (grupo 2: 75,4% no público e 97,1% no privado; grupo 4 : 55,0% no público e 88,2% na privado). Analisando a contribuição relativa dos grupos para a taxa de cesárea global, houve diferenças estatísticas para todos os grupos de gestações a termo, cefálico, sem cesárea anterior (grupos 1 a 4), enquanto que os grupos 5 e 10 contribuíram com percentuais semelhantes nos setores público e privado (Tabela 3) .

No setor público, as taxas de cesárea foram estatisticamente maiores em mulheres com alto risco obstétrico (67,7%) em comparação com mulheres de baixo risco obstétrico (35,3%). Isto era verdade para a maioria dos grupos de Robson, exceto para os grupos não-cefálicos (6 , 7 e 9 combinados). No setor privado, não houve diferenças estatisticamente significativas nas taxas de cesárea quando as mulheres de alto risco obstétrico (92,8%) foram comparadas com as mulheres de baixo risco (86,3%), com exceção do grupo 10 (Tabela 4 e Figura 1).

**DISCUSSÂO**

**Principais achados**

A taxa de cesárea no Brasil foi mais de duas vezes maior em mulheres cobertas por cuidados de saúde privado do que em mulheres que deram à luz no setor público. Os grupos com maior impacto sobre a taxa de cesárea do Brasil em ambos os setores público e privado foram o grupo 2 (nulíparas, termo, cefálica com parto induzido ou cesárea anteparto), grupo 5 (multíparas, termo, apresentação cefálica e cesárea anterior) e grupo 10 (gestações pré-termo cefálicas), que representaram mais de 70% das cesarianas realizadas no país.

A prevalência de risco obstétrico não foi diferente, apesar das discrepâncias nas características sociodemográficas das mulheres dos setores público e privado. Mulheres de alto risco obstétrico tiveram significativamente taxas de cesárea mais elevadas, quando comparadas com mulheres de baixo risco, em quase todos os grupos Robson, apenas no setor público, mas não no setor privado, o que sugere um uso liberal e excessivo da cesariana em mulheres com cuidado de saúde privado.

**Pontos fortes e limitações**

Este estudo é importante por muitas razões. Primeiro, ele foi baseado em uma pesquisa nacional, que abrange todos os estados brasileiros e que foi representativa de 2.337.475 partos (80%) que ocorreram em 2011 [19]. No nosso conhecimento, este é o terceiro estudo que utilizou a classificação Robson para avaliar as taxas de cesárea em nível nacional e o segundo a usar dados primários [21,22]. Foram coletadas todas as informações essenciais incluídas na classificação Robson, e apenas algumas mulheres não puderam ser classificadas em um dos grupos de Robson. Isso minimiza o problema da utilização de dados de rotina, que nem sempre são precisos. Em segundo lugar, estimamos idade gestacional utilizando um algoritmo, baseado principalmente na ultrassonografia obstétrica, o que confere algumas vantagens sobre a data da última menstruação, já que esta tende a superestimar a taxa de nascimentos prematuros na população brasileira [20]. Finalmente, também usamos uma definição clara para classificar as mulheres que entraram em trabalho de parto, que é comumente omissa em estudos anteriores [23].

Devido ao desenho da amostra, os resultados só podem ser extrapolados para os 80% da população que dão à luz em hospitais com mais de 500 partos por ano, e não a toda a população brasileira. Além disso, este estudo teve poder limitado para comparar as diferenças entre os setores público e privado para categorias de grupos Robson com frequência muito baixa, como as categorias 6, 7, 8, 9 e grupos de indução no setor privado (2a e 4a). Outra limitação do estudo é o potencial erro de classificação de algumas mulheres que pertenceriam aos grupos 1 e 3 e foram erroneamente classificadas nos grupos 2 e 4 por causa da definição utilizada para a indução do parto. É possível que algumas mulheres nulíparas e multíparas internadas com início espontâneo do trabalho (grupos 1 e 3) receberam ocitocina durante a fase latente, antes de chegar a 4 centímetros de dilatação, para o aceleração do trabalho de parto. No entanto, isso provavelmente não irá afetar as principais conclusões do estudo, considerando a subutilização da indução do parto neste estudo.

**Interpretação**

As taxas de cesárea continuam a aumentar em todo o mundo, sem uma compreensão clara das principais causas e suas consequências. A taxa de cesárea encontrada no estudo "Nascer no Brasil" (51,9%) está entre as mais altas do mundo, juntamente com a China (52,5%), Chipre (52,2%), República Dominicana (56,4%) e Egito (51,8%) [24-25]. Há evidências de que ela continua a crescer [6].

Nossos resultados mostraram que as mulheres que deram à luz no setor privado foram mais frequentemente brancas, mais velhas e com o ensino superior, condições associadas com a cesariana em estudos anteriores [26-27]. Embora houvesse mais mulheres multíparas, menos gestações gemelares e com cesárea anterior em mulheres no setor público, é improvável que esses fatores sozinhos possam explicar a diferença nas taxas de cesárea. O baixo uso de indução do parto no setor privado (apenas 3,5%) também foi notável, reforçando a preferência pela cesárea anteparto como forma de interrupção da gravidez. Mesmo no setor público, a taxa de partos induzidos foi menor do que em países com baixas taxas de cesárea, como a França e os Países Baixos [21,22], e também menor do que o relatado anteriormente na América Latina [28]

A presente análise das cesarianas pela classificação Robson revelou, como ocorreu em outros estudos, que o grupo de nulíparas, termo, apresentação cefálica é aquele que mais contribui para a taxa global de cesárea [21,29-30]. Analisando nove instituições, Brennan et al. [29] mostraram que 98% da variação institucional das taxas de cesárea pode ser atribuída a este grupo, que contribuiu para mais de 30% do cesarianas realizadas na França e nos Países Baixos [21,22]. Os mesmos autores também apontaram que a proporção deste grupo na população foi semelhante entre as instituições, reforçando a hipótese de que as variações nas taxas de cesárea neste grupo afetam a taxa global. No nosso estudo, a proporção dos grupos 1 e 2 em conjunto foi de 39%, semelhante à encontrada na América Latina (36,4%) [31], França (38,2%) [21], Canadá (39,7%) [30] e Países Baixos (39,9%) [22]. No entanto, no Brasil, verificamos que o grupo de cesárea anteparto (Grupo 2b) impactou mais sobre a contribuição das mulheres nulíparas a termo (14,9%). Nos países europeus, a proporção deste grupo (2b) é de cerca de 1% da população obstétrica [21,22], mas mesmo no setor público brasileiro, este grupo incluía 9% das mulheres em nosso estudo. Como o número de mulheres nulíparas é quase o mesmo, a proporção do grupo 1 (18,9%) ficou abaixo do que é comumente encontrado em outros estudos que a tem relatado acima de 25% da população obstétrica [21,22,29,31]. Quando analisamos as mulheres com pagamento privado, esse percentual era ainda menor (6,4%), apesar da maior proporção de nulíparas, termo, apresentação cefálica no setor privado (45,7%) do que no setor público (36,1%).

O grupo que isoladamente mais contribuiu para cesarianas no Brasil foi o de multíparas, termo, com cesárea anterior (grupo 5). Recentemente, uma análise da OMS constatou que as taxas de cesárea e a contribuição absoluta do grupo 5 têm aumentado nos últimos anos [16]. Estes dados mostram o efeito dominó do uso da cesariana: aumento das taxas de cesárea, especialmente em mulheres nulíparas, aumento do número de mulheres com cesárea anterior, que são mais susceptíveis a uma cesárea de repetição [16]. Como resultado da história das altas taxas de cesárea no Brasil, o grupo 5 constitui quase 20% da população do Brasil; combinado com a alta taxa de cesárea de repetição, isso torna-o responsável por quase um terço das cesáreas realizadas no país, tanto no setor público como no privado. Nossos dados são consistentes com a pesquisa “Global Survey” da OMS realizada na América Latina [31], onde o grupo 5 foi responsável ​​por 26,7% das cesarianas. A taxa de cesárea para este grupo, embora não seja diferente daquela encontrada em países com muito alto e alto índice de desenvolvimento humano nas pesquisas da OMS (de 78,1 a 79,4%) [19], é consideravelmente maior do que a encontrada na França (61%) [21] e nos Países Baixos (47%) [22]. Enquanto o sucesso do parto vaginal após cesariana (VBAC) atinge 70% em vários estudos [32], o incentivo a esta prática seria essencial para reduzir as cesarianas no Brasil. Além disso, as cesáreas de repetição aumentam a chance de placenta acreta e placenta prévia, o que pode resultar em aumento do risco em gestações subsequentes [32,33].

Os grupos de multíparas sem cesariana prévia (grupos 3 e 4) contribuíram para pouco mais de 10% das cesáreas. Digno de nota é a elevada taxa de cesárea no grupo 4 (61%), mesmo no setor público (55%), o qual está relacionado com o número de mulheres submetidas a cesárea anteparto (grupo 4b) que é maior do que aquele de submetidas a indução (4a grupo). Enquanto no Brasil o grupo 4b corresponde a 3,2% das mulheres, em outros países que não excede 1% [21,22,30]. Estes números podem, uma vez mais, a refletir a preferência pela cesárea em detrimento da indução do parto em gestações de alto risco, mas também o uso da cesariana para laqueadura tubária concomitante, como mencionado em outros estudos [8,11,34].

O terceiro grupo que mais contribuiu para a taxa de cesárea em ambos os setores foi o grupo do parto pré-termo, contribuindo para cerca de 10% das cesáreas realizada no Brasil. Este número é pouco maior que aquele encontrado em países com baixas taxas de prematuridade. Nos Países Baixos, grupo 10 corresponde a 7,1% das cesáreas [22], enquanto na França, o percentual é de 8,3% [21]. No Brasil, tanto o tamanho do grupo (9,7%) como sua taxa de cesárea (50,1%) afetaram a taxa global de cesárea.

Finalmente, os grupos de apresentação não-cefálica (grupos de 6, 7 e 9) e os gêmeos (grupo 8) contribuíram com apenas 8,9% da cesarianas. Este número é mais baixo do que o da pesquisa da OMS na América Latina (14%) [31] e consideravelmente mais baixo do que observado na França (20,5%) [21] e nos Países Baixos (27,2%) [22]. Mesmo excluindo os gêmeos, cuja prevalência nesses países é maior, e considerando apenas as apresentações não-cefálicas, a diferença permanece grande (Brasil: 7%; França: 16,5%; Países Baixos: 22,5%).

No Brasil, houve uma clara diferença tanto na distribuição das mulheres quanto nas taxas de cesárea nos grupos Robson de acordo com a fonte de pagamento. Os dois grupos com maior tamanho relativo no setor público (grupos 3 e 1) tiveram pouca importância no setor privado. Além disso, houve uma clara concentração de mulheres nulíparas no grupo 2b e multíparas no grupo 5, que representaram > 70% das cesáreas no setor privado, onde > 80% das mulheres não entraram em trabalho de parto, reforçando o ditado "uma vez cesárea, sempre cesárea".

Analisando o aumento do número de cesarianas no período entre as duas pesquisas da OMS, Vogel et al. [16], concluíram que o limiar para a indicação médica da cesariana tornou-se menor ao longo do tempo, ou houve aumento da utilização de cesárea eletiva, ou ambos ocorreram em conjunto. Este parece ser o que ocorreu no Brasil nas últimas décadas. Enquanto a taxa de cesárea é maior do que a encontrada em outros países em grupos com baixa probabilidade de cesariana (nulíparas e multíparas a termo com trabalho de parto espontâneo e multíparas com parto induzido), o uso generalizado de cesárea eletiva em mulheres nulíparas e multíparas, independentemente do risco obstétrico, mesmo no setor público, também foi observado. Na verdade, 84,2% de todas as cesarianas no Brasil são realizadas antes da fase ativa do trabalho de parto (dados não mostrados).

No setor privado, é muito provável que a cesariana não estivesse relacionada com a presença de risco obstétrico, uma vez que as taxas de cesárea, de acordo com o risco de gravidez, foram diferentes apenas no grupo 10. Além disso, as taxas de cesárea também foram extremamente elevadas em mulheres de baixo risco. Apesar das mulheres com financiamento privado terem uma maior preferência pela cesariana (36,1% nulíparas e 58,8% das multíparas no início da gravidez) [8], este fato por si só não explica essas altas taxas de cesárea.

As altas taxas de cesárea eletiva no Brasil, especialmente no setor privado, são motivo de preocupação, porque podem trazer prejuízos desnecessários para saúde das mulheres e dos bebês, se realizados sem indicação [5], incluindo o aumento da morbidade materna [3] e neonatal, especialmente quando realizadas antes de 39 semanas [35]. Nossos dados revelaram uma grande diferença nas taxas de cesárea no grupo de prematuros de baixo risco de acordo com a fonte de pagamento (25,4% do público e 71,4% privado), o que levanta questões sobre se esta prática pode estar levando a prematuridade iatrogênica.

**CONCLUSÕES**

Esta é uma análise das taxas de cesárea no Brasil pela classificação Robson usando dados de todo o país. A classificação Robson identifica os contribuintes para a taxa de cesárea, mas não fornece informações sobre as razões ou explicações para as diferenças observadas [23]. No entanto, esta classificação ajuda a identificar os grupos-alvo que podem beneficiar de implementações ou intervenções e orientar políticas públicas e investimentos para reduzir as taxas de cesárea no Brasil.

As políticas públicas devem ser dirigidas para redução da cesariana nas nulíparas, especialmente através da redução do número de cesáreas eletivas nestas mulheres. O uso alargado da indução do parto e sua condução adequada em detrimento da cesárea anteparto seria uma medida importante para reduzir as taxas de cesárea. Encorajar o VBAC e reduzir a cesárea de repetição são igualmente importantes, uma vez que > 70% das cesarianas realizadas no país ocorreram nestes grupos. Estas políticas também devem ser dirigidas para o setor privado, onde é realizado um terço de todas as cesarianas do Brasil e onde a indicação da cirurgia não parece ser motivada por razões médicas.

**Conflito de interesses**

Os autores declaram que não têm conflito de interesses. Os financiadores do estudo não tiveram nenhum papel no desenho do estudo, coleta de dados, análise de dados, interpretação de dados, ou na realização do artigo.

**Contribuições dos autores**

MNP e MCL foram responsáveis pela concepção e desenho do estudo. MNP e APEP fizeram a análise dos dados. MNP escreveu o primeiro rascunho do manuscrito e incorporou contribuições substanciais de MCL, APEP, RMSMD, JAT, MABD e MEM. Todos os autores aprovaram a versão final.

**Agradecimentos**

Este trabalho foi apoiado pelo financiamento do Conselho Nacional de Desenvolvimento Científico e Tecnológico (CNPq); da Escola Nacional de Saúde Pública, Fundação Oswaldo Cruz (INOVA Projeto); e da Agência de Financiamento à Pesquisa do Estado do Rio de Janeiro (FAPERJ).

**Referências**

1. World Health Organization. WHO statement on caesarean section rates. 2015. <http://apps.who.int/iris/bitstream/10665/161442/1/WHO_RHR_15.02_eng.pdf?ua=1> Accessed 14 jun 2015.
2. Deneux-Tharaux C, Carmona E, Bouvier-Colle MH, Bréart G. [Postpartum maternal mortality and cesarean delivery.](http://www.ncbi.nlm.nih.gov/pubmed/16946213?ordinalpos=8&itool=EntrezSystem2.PEntrez.Pubmed.Pubmed_ResultsPanel.Pubmed_DefaultReportPanel.Pubmed_RVDocSum) Obstet Gynecol 2006;108:541-8.
3. Liu S, Liston RM, Joseph KS, Heaman M, Sauve R, Kramer MS *et al.* [Maternal mortality and severe morbidity associated with low-risk planned cesarean delivery versus planned vaginal delivery at term.](http://www.ncbi.nlm.nih.gov/pubmed/17296957?ordinalpos=20&itool=EntrezSystem2.PEntrez.Pubmed.Pubmed_ResultsPanel.Pubmed_DefaultReportPanel.Pubmed_RVDocSum) CMAJ 2007; 176:455-60.
4. Villar J, Carroli G, Zavaleta N, Donner A, Wojdyla D, Faundes A, *et al.* [Maternal and neonatal individual risks and benefits associated with caesarean delivery: multicentre prospective study.](http://www.ncbi.nlm.nih.gov/pubmed/17977819?ordinalpos=34&itool=EntrezSystem2.PEntrez.Pubmed.Pubmed_ResultsPanel.Pubmed_DefaultReportPanel.Pubmed_RVDocSum) BMJ 2007; 335: 1025.
5. Souza JP, Gülmezoglu A, Lumbiganon P, Laopaiboon M, Carroli G, Fawole B, *et al.* [Caesarean section without medical indications is associated with an increased risk of adverse short-term maternal outcomes: the 2004-2008 WHO Global Survey on Maternal and Perinatal Health.](http://www.ncbi.nlm.nih.gov/pubmed/21067593) BMC Med. 2010;8:71.
6. Sistema de Informações sobre Nascidos Vivos. DATASUS. Ministério da Saúde. 2016. <http://www.datasus.gov.br> Accessed 16 mar 2016.
7. Victora CG, Aquino EM, do Carmo Leal M, Monteiro CA, Barros FC, Szwarcwald CL. [Maternal and child health in Brazil: progress and challenges.](http://www.ncbi.nlm.nih.gov/pubmed/21561656) Lancet. 2011;377(9780):1863-76.
8. Domingues RM, Dias MA, Nakamura-Pereira M, Torres JA, d'Orsi E, Pereira AP, *et al.* [Process of decision-making regarding the mode of birth in Brazil: from the initial preference of women to the final mode of birth.](http://www.ncbi.nlm.nih.gov/pubmed/25167169) Cad Saude Publica. 2014;30 Suppl 1:S101-116.
9. Hopkins K, de Lima Amaral EF, Mourão AN. [The impact of payment source and hospital type on rising cesarean section rates in Brazil, 1998 to 2008.](http://www.ncbi.nlm.nih.gov/pubmed/24684250) Birth. 2014;41(2):169-77.
10. Ramires de Jesus G, Ramires de Jesus N, Peixoto-Filho FM, Lobato G. [Caesarean rates in Brazil: what is involved?](http://www.ncbi.nlm.nih.gov/pubmed/25327984) BJOG. 2015;122(5):606-9.
11. Hopkins K. [Are Brazilian women really choosing to deliver by cesarean?](http://www.ncbi.nlm.nih.gov/pubmed/10975232) Soc Sci Med. 2000;51(5):725-40.
12. Dias MA, Domingues RM, Pereira AP, Fonseca SC, da Gama SG, Theme Filha MM, *et al.* [[The decision of women for cesarean birth: a case study in two units of the supplementary health care system of the State of Rio de Janeiro].](http://www.ncbi.nlm.nih.gov/pubmed/18813654) Cien Saude Colet. 2008;13(5):1521-34.
13. Carmo Leal M, Pereira AP, Domingues RM, Theme Filha MM, Dias MA, Nakamura-Pereira M et al. Obstetric interventions during labor and childbirth in Brazilian low-risk women. Cad Saude Publica 2014;30 Suppl 1:S17-32.
14. Robson MS. [Can we reduce the caesarean section rate?](http://www.ncbi.nlm.nih.gov/pubmed/11359322) Best Pract Res Clin Obstet Gynaecol. 2001; 15(1):179-94.
15. Torloni MR, Betran AP, Souza JP, Widmer M, Allen T, Gulmezoglu M, Merialdi M. [Classifications for cesarean section: a systematic review.](http://www.ncbi.nlm.nih.gov/pubmed/21283801) PLoS One. 2011; 6(1):e14566.
16. Vogel JP, Betrán AP, Vindevoghel N, Souza JP, Torloni MR, Zhang J, *et al.* [Use of the Robson classification to assess caesarean section trends in 21 countries: a secondary analysis of two WHO multicountry surveys.](http://www.ncbi.nlm.nih.gov/pubmed/25866355) Lancet Glob Health. 2015;3(5):e260-70.
17. do Carmo Leal M, da Silva AA, Dias MA, da Gama SG, Rattner D, Moreira ME et al. Birth in Brazil: national survey into labour and birth. Reproductive health. 2012;9:15.
18. Azevedo Bittencourt SD, Costa Reis LG, Ramos MM, Rattner D, Rodrigues PL, Neves DC, et al. [Structure in Brazilian maternity hospitals: key characteristics for quality of obstetric and neonatal care.](http://www.ncbi.nlm.nih.gov/pubmed/25167180) Cad Saude Publica. 2014; 30 Suppl 1:S208-19.
19. Vasconcellos MT, Silva PL, Pereira AP, Schilithz AO, Souza Junior PR, Szwarcwald CL. Sampling design for the Birth in Brazil: National Survey into Labor and Birth. Cad Saude Publica. 2014;30 Suppl 1:S49-58.
20. Pereira AP, Leal Mdo C, da Gama SG, Domingues RM, Schilithz AO, Bastos MH. Determining gestational age based on information from the Birth in Brazil study. Cad Saude Publica. 2014;30 Suppl 1:S59-70.
21. Le Ray C, Blondel B, Prunet C, Khireddine I, Deneux-Tharaux C, Goffinet F. [Stabilising the caesarean rate: which target population?](http://www.ncbi.nlm.nih.gov/pubmed/25412695) BJOG. 2015;122(5):690-9.
22. Zhang J, Geerts C, Hukkelhoven C, Offerhaus P, Zwart J, de Jonge A. [Caesarean section rates in subgroups of women and perinatal outcomes.](http://www.ncbi.nlm.nih.gov/pubmed/26216434) BJOG. 2016; 123(5):754-61.
23. Betrán AP, Vindevoghel N, Souza JP, Gülmezoglu AM, Torloni MR. [A systematic review of the Robson classification for caesarean section: what works, doesn't work and how to improve it.](http://www.ncbi.nlm.nih.gov/pubmed/24892928) PLoS One. 2014;9(6):e97769.
24. Global Health Observatory Data Repository. World Health Organization. 2015 <http://apps.who.int/gho/data/node.main.REPWOMEN39?lang=en> Accessed 14 jun 2015.
25. Hellerstein S, Feldman S, Duan T. [China's 50% caesarean delivery rate: is it too high?](http://www.ncbi.nlm.nih.gov/pubmed/25138909) BJOG. 2015;122(2):160-4.
26. Rebelo F, da Rocha CM, Cortes TR, Dutra CL, Kac G. [High cesarean prevalence in a national population-based study in Brazil: the role of private practice.](http://www.ncbi.nlm.nih.gov/pubmed/20583936) Acta Obstet Gynecol Scand. 2010;89(7):903-8.
27. Barros AJ, Santos IS, Matijasevich A, Domingues MR, Silveira M, Barros FC, et al. [Patterns of deliveries in a Brazilian birth cohort: almost universal cesarean sections for the better-off.](http://www.ncbi.nlm.nih.gov/pubmed/21670862) Rev Saude Publica. 2011;45(4):635-43.
28. Guerra GV, Cecatti JG, Souza JP, Faúndes A, Morais SS, Gülmezoglu AM, et al. [Factors and outcomes associated with the induction of labour in Latin America.](http://www.ncbi.nlm.nih.gov/pubmed/19906020) BJOG. 2009;116(13):1762-72.
29. Brennan DJ, Robson MS, Murphy M, O'Herlihy C. [Comparative analysis of international cesarean delivery rates using 10-group classification identifies significant variation in spontaneous labor.](http://www.ncbi.nlm.nih.gov/pubmed/19733283) Am J Obstet Gynecol. 2009; 201(3):308.e1-8.
30. Kelly S, Sprague A, Fell DB, Murphy P, Aelicks N, Guo Y, et al. [Examining caesarean section rates in Canada using the Robson classification system.](http://www.ncbi.nlm.nih.gov/pubmed/23470108) J Obstet Gynaecol Can. 2013;35(3):206-14.
31. Betrán AP, Gulmezoglu AM, Robson M, Merialdi M, Souza JP, Wojdyla D, *et al*. [WHO global survey on maternal and perinatal health in Latin America: classifying caesarean sections.](http://www.ncbi.nlm.nih.gov/pubmed/19874598) Reprod Health. 2009;6:18.
32. Guise JM, Eden K, Emeis C, Denman MA, Marshall N, Fu RR, Janik R, Nygren P, Walker M, McDonagh M. [Vaginal birth after cesarean: new insights.](http://www.ncbi.nlm.nih.gov/pubmed/20629481) Evid Rep Technol Assess (Full Rep). 2010;(191):1-397.
33. Silver RM, Landon MB, Rouse DJ, Leveno KJ, Spong CY, Thom EA, et al. [Maternal morbidity associated with multiple repeat cesarean deliveries.](http://www.ncbi.nlm.nih.gov/pubmed/16738145) Obstet Gynecol. 2006;107(6):1226-32.
34. Potter EJ, Berquó E, Perpetuo IHO, Leal OF, Hopkins K, Souza MR, et al. Unwanted caesarean sections among public and private patients in Brazil: prospective study. BMJ 2001; 323:1155-8.
35. Tita AT, Landon MB, Spong CY, Lai Y, Leveno KJ, Varner MW, et al. [Timing of elective repeat cesarean delivery at term and neonatal outcomes.](http://www.ncbi.nlm.nih.gov/pubmed/19129525) N Engl J Med. 2009;360(2):111-20.

**Legenda das figuras**

Figura 1: Taxas de cesárea nos grupos de Robson de acordo com o risco obstétrico (mulheres de baixo risco^1^ e mulheres de alto risco^2^) nos setores público e privado.

1 mulheres sem nenhuma característica de alto risco

2 doenças hipertensivas, eclâmpsia, diabetes pré-gestacional, diabetes gestational, doenças crônicas graves, infecção na internação hospitalar para o parto, descolamento prematuro da placenta, placenta prévia, crescimento intrauterino restrito e malformações maiores do recém-nascido

**Tabelas**

| **Tabela 1 – Características das mulheres de acordo coma fonte de pagamento para o parto. Nascer no Brasil, 2011-2012** | | | | | |  |  |
| --- | --- | --- | --- | --- | --- | --- | --- |
|  | Total | | Público | | Privado | | Chi quadrado P-valor* |
|  | n | % | n | % | n | % |  |
| Total | 23,894 | | 19,129 | | 4,765 | | - |
| **Idade materna** |  |  |  |  |  |  |  |
| < 20 | 4,571 | 19.1 | 4,325 | 22.6 | 246 | 5.2 | <0.001 |
| 20-34 | 16,807 | 70.4 | 13,162 | 68.8 | 3,645 | 76.5 |  |
| > 34 | 2,509 | 10.5 | 1,635 | 8.6 | 874 | 18.3 |  |
| **Cor da pele** |  |  |  |  |  |  |  |
| Branca | 8,078 | 33.8 | 5,484 | 28.7 | 2,594 | 54.4 | <0.001 |
| Preta | 2,051 | 8.6 | 1,892 | 9.9 | 159 | 3.3 |  |
| Parda | 13,404 | 56.1 | 11,457 | 59.9 | 1,947 | 40.9 |  |
| Amarela | 257 | 1.1 | 202 | 1.1 | 55 | 1.2 |  |
| Indígena | 99 | 0.4 | 89 | 0.5 | 10,0 | 0.2 |  |
| **Estado marital** |  |  |  |  |  |  |  |
| Moro com parceiro | 19,440 | 81.4 | 15,177 | 79.4 | 4,263 | 89.5 | <0.001 |
| Não mora com parceiro | 4,431 | 18.6 | 3,931 | 20.6 | 500 | 10.5 |  |
| **Anos de escolaridade** |  |  |  |  |  |  |  |
| ≤ 7 | 6,363 | 26.5 | 6,197 | 32.4 | 166 | 3.5 | <0.001 |
| 8 to 10 | 6,104 | 25.6 | 5,604 | 29.3 | 500 | 10.5 |  |
| 11 to 14 | 9,310 | 39.0 | 6,790 | 35.5 | 2,520 | 52.9 |  |
| ≥ 15 | 2,112 | 8.9 | 535 | 2.8 | 1,577 | 33.1 |  |
| **Paridade** |  |  |  |  |  |  |  |
| 0 | 11,208 | 46.9 | 8,569 | 44.8 | 2,639 | 55.4 | <0.001 |
| 1 | 7,015 | 29.4 | 5,405 | 28.3 | 1,610 | 33.8 |  |
| ≥2 | 5,671 | 23.7 | 5,155 | 26.9 | 516 | 10.8 |  |
| **Cesarianas prévias**** |  |  |  |  |  |  |  |
| 0 | 7,571 | 59.7 | 6,885 | 65.2 | 686 | 32.2 | <0.001 |
| 1 | 3,905 | 30.8 | 2,689 | 25.5 | 1,216 | 57.2 |  |
| ≥ 2 | 1,211 | 9.5 | 986 | 9.3 | 225 | 10.6 |  |
| **Tipo de gestação** |  |  |  |  |  |  |  |
| Única | 23,610 | 98.8 | 18,936 | 99.0 | 4,674 | 98.1 | <0.001 |
| Múltipla | 284 | 1.2 | 192 | 1.0 | 92 | 1.9 |  |
| **Indução do parto** |  |  |  |  |  |  |  |
| Sim | 2,729 | 11.4 | 2,561 | 13.4 | 168 | 3.5 | <0.001 |
| Não | 21,165 | 88.6 | 16,568 | 86.6 | 4,597 | 96.5 |  |
| **Trabalho de parto (espontâneo ou induzido)** |  |  |  |  |  |  |  |
| Sim | 13,458 | 56.3 | 12,618 | 66.0 | 840 | 17.6 | <0.001 |
| Não | 10,436 | 43.7 | 6,511 | 34.0 | 3,925 | 82.4 |  |
| **Parto** |  |  |  |  |  |  |  |
| Vaginal | 11,152 | 46.7 | 10,605 | 55.4 | 547 | 11.5 | <0.001 |
| Forceps/Vácuo | 347 | 1.5 | 317 | 1.7 | 30 | 0.6 |  |
| Cesárea | 12,395 | 51.9 | 8,207 | 42.9 | 4,188 | 87.9 |  |
| **Alto risco obstétrico***** |  |  |  |  |  |  |  |
| Sim | 5,677 | 23.8 | 4,487 | 23.5 | 1,190 | 25.0 | 0.225 |
| Não | 18,217 | 76.2 | 14,642 | 76.5 | 3,575 | 75.0 |  |
| * χ2 teste. |  |  |  |  |  |  |  |
| ** Somente mulheres com parto anterior | |  |  |  |  |  |  |
| *** doenças hipertensivas, eclâmpsia, diabetes pré-gestacional, diabetes gestational, doenças crônicas graves, infecção na internação hospitalar para o parto, descolamento prematuro da placenta, placenta prévia, crescimento intrauterino restrito e malformações maiores do recém-nascido | | | | | | | |

| **Tabela 2 – Classificação de Robson na pesquisa Nascer no Brasil, 2011-2012** | | | | | | | | |
| --- | --- | --- | --- | --- | --- | --- | --- | --- |
| Grupo de Robson | Descrição da população obstétrica | Número de cesarianas | Número de nascimentos | | Tamanho relativo (%) do grupo^1^ | Taxa de cesárea (%) em cada grupo | Contribuição absoluta (%) na taxa global de cesárea^2^ | Contribuição relativa (%) na taxa global de cesárea^3^ |
| 1 | Mulheres nulíparas, única, cefálica, >=37 semanas, trabalho de parto espontâneo | 848 | 4,330 | | 18.1 | 19.6 | 3.5 | 6.8 |
| 2 | Mulheres nulíparas, única, cefálica, >=37 weeks, induzido ou cesárea anteparto | 4,169 | 4,988 | | 20.9 | 83.6 | 17.4 | 33.6 |
| 2a | Mulheres nulíparas, única, cefálica, >=37 weeks, parto induzido | 618 | 1,437 | | 6.0 | 43.0 | 2.6 | 5.0 |
| 2b | Mulheres nulíparas, única, cefálica, >=37 weeks, cesárea anteparto | 3,551 | 3,551 | | 14.9 | 100.0 | 14.9 | 28.6 |
| 3 | Mulheres multíparas (excluindo cesárea prévia), única, cefálica, >=37 semanas, trabalho de parto espontâneo | 264 | 4,775 | | 20.0 | 5.5 | 1.1 | 2.1 |
| 4 | Mulheres multíparas sem cesárea anterior, com gestação única cefálica, >=37 semanas, induzido ou cesárea anteparto | 1,028 | 1,685 | | 7.1 | 61.0 | 4.3 | 8.3 |
| 4a | Mulheres multíparas sem cesárea anterior, com gestação única cefálica, >=37 semanas, parto induzido | 127 | 784 | | 3.3 | 16.2 | 0.5 | 1.0 |
| 4b | Mulheres multíparas sem cesárea anterior, com gestação única cefálica, >=37 semanas, cesárea anteparto | 901 | 901 | | 3.8 | 100.0 | 3.8 | 7.3 |
| 5 | Cesárea anterior, única, cefálica, >=37 semanas | 3,816 | 4,562 | | 19.1 | 83.6 | 16.0 | 30.8 |
| 6 | Todas nulíparas com pélvico | 409 | 425 | | 1.8 | 96.2 | 1.7 | 3.3 |
| 7 | Todas multíparas com pélvico (incluindo cesárea anterior) | 338 | 399 | | 1.7 | 84.7 | 1.4 | 2.7 |
| 8 | Todas gestações múltiplas (incluindo cesárea anterior) | 240 | 283 | | 1.2 | 84.8 | 1.0 | 1.9 |
| 9 | Todas apresentações anômalas (incluindo cesárea anterior) | 114 | 114 | | 0.5 | 100.0 | 0.5 | 0.9 |
| 10 | Todas gestações únicas, cefálicas, <=36 semanas (incluindo cesárea anterior) | 1,166 | 2,326 | | 9.7 | 50.1 | 4.9 | 9.4 |
| X | Sem classificação | 3 | 7 | | 0.0 | 42.9 | 0,0 | 0.0 |
|  | Total | 12,395 | 23,894 | | 100 | 51.9 | 51.9 | 100 |
| 1 (Número de nascimentos no grupo) / (número total de nascimentos). | | | |  |  |  |  |  |
| 2 (Número de cesarianas no grupo) / (número total de nascimentos) | | | |  |  |  |  |  |
| 3 (Número de cesarianas no grupo) / (número total de cesarianas) | | | |  |  |  |  |  |

| **Tabela 3 - Grupo de Robson de acordo com a fonte de pagamento para o parto. Nascer no Brasil, 2011-2012** | | | | | | | | | | | | | | | | | | | | | | | | | | | | | | | | | |  |  |  |  |  |  |  |  |  |
| --- | --- | --- | --- | --- | --- | --- | --- | --- | --- | --- | --- | --- | --- | --- | --- | --- | --- | --- | --- | --- | --- | --- | --- | --- | --- | --- | --- | --- | --- | --- | --- | --- | --- | --- | --- | --- | --- | --- | --- | --- | --- | --- |
| Grupo de Robson | Descrição da população obstétrica | Número de cesarianas | | Número de nascimentos | | Tamanho relativo (%) do grupo^1,a^ | | | | | | Taxa de cesárea (%) em cada grupo^2,b^ | | | | | | | | | Contribuição relativa (%) na taxa global de cesárea^3,c^ | | | | | | | | |  |  |  |  |  |  |  |  |  |  |  |  |  |
|  |  | Público | Privado | Público | Privado | Público | | Privado | | | Público | | | Privado | | | | | Público | | | | | | Privado | | | | |  |  |  |  |  |  |  |  |  |  |  |  |  |
|  |  |  |  |  |  | % | IC 95% | | % | IC 95% | | % | IC 95% | | % | IC 95% | | | | % | | | IC 95% | | | % | | IC 95% | | |  |  |  |  |  |  |  |  |  |  |  |  |
| 1 | Mulheres nulíparas, única, cefálica, >=37 semanas, trabalho de parto espontâneo | 712 | 136 | 4,023 | 307 | **21.0** | **(19.5 - 22.2)** | | **6.4** | **(5.1 - 7.7)** | | **17.7** | **(15.1 - 20.6)** | | **44.4** | **(35.5 - 53.5)** | | | | **8.7** | | | **(7.5 - 10.1)** | | | **3.2** | | **(2.6 - 4.1)** | | |  |  |  |  |  |  |  |  |  |  |  |  |
| 2 | Mulheres nulíparas, única, cefálica, >=37 weeks, induzido ou cesárea anteparto | 2,351 | 1,818 | 3,116 | 1,872 | **16.3** | **(15.2 - 17.1)** | | **39.3** | **(36.2 - 40.7)** | | **75.4** | **(71.2 - 79.2)** | | **97.1** | **(93.3 - 98.8)** | | | | **28.6** | | | **(26.9 - 30.4)** | | | **43.4** | | **(41.2 - 45.7)** | | |  |  |  |  |  |  |  |  |  |  |  |  |
| 2ª | Mulheres nulíparas, única, cefálica, >=37 weeks, parto induzido | 566 | 52 | 1,331 | 106 | **7.0** | **(6.1 - 8.0)** | | **2.2** | **(1.3 - 3.7)** | | 42.5 | (36.8 - 48.4) | | 49.1 | (29.6 - 69.1) | | | | **6.9** | | | **(5.7 - 8.3)** | | | **1.2** | | **(0.8 - 1.9)** | | |  |  |  |  |  |  |  |  |  |  |  |  |
| 2b | Mulheres nulíparas, única, cefálica, >=37 weeks, cesárea anteparto | 1,785 | 1,766 | 1,785 | 1,766 | **9.3** | **(8.5 - 10.3)** | | **37.1** | **(34.5 - 39.7)** | | 100.0 | - | | 100.0 | - | | | | **21.8** | | | **(20.3 - 23.3)** | | | **42.2** | | **(39.9 - 44.4)** | | |  |  |  |  |  |  |  |  |  |  |  |  |
| 3 | Mulheres multíparas (excluindo cesárea prévia), única, cefálica, >=37 semanas, trabalho de parto espontâneo | 234 | 30 | 4,520 | 255 | **23.6** | **(22.3 - 24.6)** | | **5.4** | **(4.0 - 6.9)** | | **5.2** | **(4.1 - 6.5)** | | **11.8** | **(7.2 - 18.1)** | | | | **2.9** | | | **(2.3 - 3.5)** | | | **0.7** | | **(0.5 - 1.0)** | | |  |  |  |  |  |  |  |  |  |  |  |  |
| 4 | Mulheres multíparas sem cesárea anterior, com gestação única cefálica, >=37 semanas, induzido ou cesárea anteparto | 758 | 270 | 1,379 | 306 | 7.2 | (6.5 - 7.8) | | 6.4 | (5.3 - 7.4) | | **55.0** | **(50.0 - 59.8)** | | **88.2** | **(80.1 - 93.2)** | | | | **9.2** | | | **(8.4 - 10.1)** | | | **6.4** | | **(5.4 - 7.6)** | | |  |  |  |  |  |  |  |  |  |  |  |  |
| 4ª | Mulheres multíparas sem cesárea anterior, com gestação única cefálica, >=37 semanas, parto induzido | 118 | 9 | 739 | 45 | **3.9** | **(3.3 - 4.5)** | | **0.9** | **(0.6 - 1.6)** | | 16.0 | (13.0 - 19.6) | | 19.5 | (8.7 - 38.1) | | | | **1.4** | | | **(1.1 - 1.8)** | | | **0.2** | | **(0.1 - 0.5)** | | |  |  |  |  |  |  |  |  |  |  |  |  |
| 4b | Mulheres multíparas sem cesárea anterior, com gestação única cefálica, >=37 semanas, cesárea anteparto | 640 | 261 | 640 | 261 | **3.3** | **(3.0 - 3.8)** | | **5.5** | **(4.5 - 6.6)** | | 100.0 | - | | 100.0 | - | | | | 7.8 | | | (7.0 - 8.6) | | | 6.2 | | (5.2 - 7.4) | | |  |  |  |  |  |  |  |  |  |  |  |  |
| 5 | Cesárea anterior, única, cefálica, >=37 semanas | 2,556 | 1,260 | 3,276 | 1,286 | **17.1** | **(16.0 - 18.0)** | | **27.0** | **(24.8 - 28.1)** | | **78.0** | **(75.4 - 80.4)** | | **98.0** | **(96.5 - 98.9)** | | | | 31.1 | | | (29.7 - 32.7) | | | 30.1 | | (28.5 - 31.8) | | |  |  |  |  |  |  |  |  |  |  |  |  |
| 6 | Todas nulíparas com pélvico | 271 | 138 | 286 | 139 | **1.5** | **(1.3 - 1.7)** | | **2.9** | **(2.0 - 4.0)** | | **94.4** | **(91.2 - 96.9)** | | **99.3** | **(96.5 - 99.9)** | | | | 3.3 | | | (2.8 - 3.8) | | | 3.3 | | (2.3 - 4.6) | | |  |  |  |  |  |  |  |  |  |  |  |  |
| 7 | Todas multíparas com pélvico (incluindo cesárea anterior) | 276 | 62 | 336 | 63 | 1.8 | (1.5 - 2.1) | | 1.3 | (1.0 - 1.7) | | **82.1** | **(71.9 - 89.1)** | | **98.4** | **(93.0 - 99.6)** | | | | **3.4** | | | **(2.9 - 3.9)** | | | **1.5** | | **(1.2 - 1.9)** | | |  |  |  |  |  |  |  |  |  |  |  |  |
| 8 | Todas gestações múltiplas (incluindo cesárea anterior) | 153 | 87 | 191 | 92 | **1.0** | **(0.8 - 1.2)** | | **1.9** | **(1.5 - 2.6)** | | 79.7 | (72.1 - 85.6) | | 94.6 | (72.7 - 99.1) | | | | 1.9 | | | (1.5 - 2.3) | | | 2.1 | | (1.5 - 3.0) | | |  |  |  |  |  |  |  |  |  |  |  |  |
| 9 | Todas apresentações anômalas (incluindo cesárea anterior) | 91 | 23 | 91 | 23 | 0.5 | (0.4 - 0.6) | | 0.5 | (0.3 - 0.8) | | 100.0 | - | | 100.0 | - | | | | **1.1** | | | **(0.9 - 1.4)** | | | **0.5** | | **(0.3 - 0.9)** | | |  |  |  |  |  |  |  |  |  |  |  |  |
| 10 | Todas gestações únicas, cefálicas, <=36 semanas (incluindo cesárea anterior) | 803 | 363 | 1,904 | 422 | 10.0 | (8.7 - 11.2) | | 8.9 | (7.1 - 10.6) | | **42.2** | **(38.7 - 45.7)** | | **86.0** | **(78.3 - 91.1)** | | | | 9.8 | | | (8.3 - 11.5) | | | 8.7 | | (6.9 - 10.9) | | |  |  |  |  |  |  |  |  |  |  |  |  |
| X | Sem classificação | 2 | 1 | 6 | 1 | 0.03 | (0.01 - 0.07) | | 0.02 | (0.002 -0.01) | | 40.2 | (7.8 - 84.2) | | 100.0 | - | | | | 0.0 | | | - | | | 0.0 | | - | | |  |  |  |  |  |  |  |  |  |  |  |  |
|  | Total deliveries | 8,207 | 4,188 | 19,128 | 4,766 | 100.0 | - | | 100.0 | - | | 41.5 | - | | 87.9 | - | | | | 100.0 | | |  | | | 100.0 | |  | | |  |  |  |  |  |  |  |  |  |  |  |  |
| 1 (Número de nascimentos no grupo) / (número total de nascimentos). | | | | | | | | | | | | | | | |  |  | | | |  | |  | | |  | |  | | |  |  | |  |  |  |  |  |  |  |  | |
| 2 (Número de cesarianas) / (número de nascimentos nos mesmo grupo de Robson). | | | | | | | | | | | | | | | |  |  | | | |  | |  | | |  | |  | | |  |  | |  |  |  |  |  |  |  |  | |
| 3 (Número de cesarianas no grupo) / (número total de cesarianas) | | | | | | | | | | | | | | | |  |  | | | |  | |  | | |  | |  | | |  |  | |  |  |  |  |  |  |  |  | |
| a As proporções Público vs.Privado diferem significativamente ao nível de .05 por meio do z-teste com ajuste de Bonferroni para todos os grupos de Robson, exceto os grupos 4, 7, 9, 10 e X. | | | | | | | | | | | | | | | | | | | | | | | | | | | | | | | | | | | | | | | | | |  |
| b As proporções Público vs.Privado diferem significativamente ao nível de .05 por meio do z-teste com ajuste de Bonferroni para todos os grupos de Robson, exceto os grupos 2a, 4a and 8. | | | | | | | | | | | | | | | | | | | | | | | | | | | | | | | | | | | | | | | | | |  |
| c As proporções Público vs.Privado diferem significativamente ao nível de .05 por meio do z-teste com ajuste de Bonferroni para todos os grupos de Robson, exceto os grupos 4b, 5, 6, 8 and 10. | | | | | | | | | | | | | | | | | | | | | | | | | | | | | | | | | | | | | | | | | |  |

| **Table 4 – Taxas de cesárea (%) por grupo de Robson em mulheres de alto e baixo risco de acordo com a fonte de pagamento para o parto. Nascer no Brasil, 2011-2012** | | | | | | | | | | | | | | | | | | | | |
| --- | --- | --- | --- | --- | --- | --- | --- | --- | --- | --- | --- | --- | --- | --- | --- | --- | --- | --- | --- | --- |
| Grupo de Robson |  |  | **Público** | | | | | | | |  | **Privado** | | | | | | | | |
|  |  | Mulheres de baixo risco^1^ | | | |  | Mulheres de alto risco^2^ | | | |  | Mulheres de baixo risco^1^ | | | |  |  | Mulheres de alto risco^2^ | | |
|  |  | Todas | Cesáreas | % | IC 95%^a^ |  | Todas | Cesáreas | % | IC 95%^a^ |  | Todas | Cesáreas | % | IC 95%^b^ |  | Todas | Cesáreas | % | IC 95%^b^ |
| Todos os grupos |  | 14,640 | 5,168 | **35.3** | (33.0 - 37.6) |  | 4,484 | 3,036 | **67.7** | (64.9 - 70.4) |  | 3,573 | 3,082 | 86.3 | (81.2 - 90.1) |  | 1,190 | 1,104 | 92.8 | (89.5 - 95.0) |
| 1 |  | 3,475 | 477 | **13.7** | (11.2 - 16.6) |  | 550 | 236 | **42.9** | (36.8 - 49.2) |  | 253 | 110 | 43.4 | (33.8 - 53.4) |  | 54 | 26 | 48.7 | (29.9 - 67.8) |
| 2 |  | 2,114 | 1,465 | **69.3** | (63.9 - 74.2) |  | 1,002 | 885 | **88.4** | (85.2 - 91.0) |  | 1,425 | 1,376 | 96.6 | (91.4 - 98.7) |  | 444 | 441 | 99.0 | (96.9 - 99.7) |
| 3 |  | 3,886 | 137 | **3.5** | (2.6 - 4.7) |  | 636 | 97 | **15.3** | (11.2 - 20.4) |  | 214 | 23 | 10.7 | (6.5 - 17.2) |  | 41 | 7 | 16.1 | (7.6 - 30.9) |
| 4 |  | 911 | 434 | **47.6** | (42.1 - 53.2) |  | 467 | 324 | **69.3** | (62.9 - 75.1) |  | 220 | 192 | 87.3 | (77.7 - 93.1) |  | 85 | 77 | 90.4 | (82.2 - 95.0) |
| 5 |  | 2,410 | 1,799 | **74.7** | (71.3 - 77.8) |  | 866 | 756 | **87.3** | (84.3 - 89.7) |  | 1,002 | 978 | 97.6 | (95.7 - 98.7) |  | 284 | 283 | 99.6 | (97.5 - 100.0) |
| 8 |  | 125 | 95 | **75.7** | (66.4 - 83.1) |  | 67 | 59 | **88.6** | (79.6 - 93.9) |  | 67 | 62 | 92.3 | (63.0 - 98.8) |  | 25 | 25 | 100.0 | - |
| 10 |  | 1,208 | 306 | **25.4** | (21.8 - 29.2) |  | 694 | 497 | **71.4** | (65.4 - 76.8) |  | 222 | 172 | **77.6** | (67.5 - 85.3) |  | 201 | 190 | **94.9** | **(88.3 - 97.9)** |
| 6, 7 e 9 |  | 511 | 455 | 89.1 | (81.4 - 93.9) |  | 202 | 182 | 90.3 | (83.0 - 94.6) |  | 170 | 169 | 99.4 | (97.4 - 99.9) |  | 56 | 55 | 98.7 | (91.4 - 99.8) |
| 1 mulheres sem características de alto risco | | | | | | | | | | | | | | | | | | | |  |
| 2 doenças hipertensivas, eclâmpsia, diabetes pré-gestacional, diabetes gestational, doenças crônicas graves, infecção na internação hospitalar para o parto, descolamento prematuro da placenta, placenta prévia, crescimento intrauterino restrito e malformações maiores do recém-nascido | | | | | | | | | | | | | | | | | | | | |
|  |  |  |  |  |  |  |  |  |  |  |  |  |  |  |  |  |  |  |  |  |
| a Proporções de cesarianas de mulheres de baixo risco vs. mulheres de alto risco diferem significativamente ao nível de .05 level por meio do z-teste com ajuste de Bonferroni para todos os grupos de Robson, exceto os grupos 6, 7 and 9 combinados. | | | | | | | | | | | | | | | | | | | | |
| b Proporções de cesarianas de mulheres de baixo risco vs. mulheres de alto risco **NÃO** diferem significativamente ao nível de .05 level por meio do z-teste com ajuste de Bonferroni para todos os grupos de Robson, exceto o grupo 10. | | | | | | | | | | | | | | | | | | | | |

| **Tabela adicional 1 – Características das instituições de acordo com o tipo de financiamento.**  **Nascer no Brasil, 2011-2012** | | | | | | |
| --- | --- | --- | --- | --- | --- | --- |
|  | Público ou Misto* | | Privado | |  |  |
|  | n | % | n | % |  |  |
| **Total** | **218** | **100** | **48** | **100** |  |  |
| **Partos por ano** |  |  |  |  |  |  |
| ≥ 3000 | 38 | 17,5 | 4 | 8,7 |  |  |
| 1000 to 2999 | 112 | 51,3 | 23 | 48,8 |  |  |
| ≤ 999 | 68 | 31,2 | 21 | 42,5 |  |  |
| **UTI (materna ou neonatal)** |  |  |  |  |  |  |
| Sim | 124 | 56,9 | 42 | 87,5 |  |  |
| Não | 94 | 43,1 | 6 | 12,5 |  |  |
| **UTI neonatal** |  |  |  |  |  |  |
| Sim | 86 | 39,2 | 38 | 79,2 |  |  |
| Não | 132 | 60,8 | 10 | 20,8 |  |  |
| **UTI materna** |  |  |  |  |  |  |
| Sim | 113 | 51.8 | 36 | 74.9 |  |  |
| Não | 105 | 48.2 | 12 | 25.1 |  |  |
| **Ventilador mecânico (adulto)** |  |  |  |  |  |  |
| Disponível | 181 | 83,0 | 47 | 98,8 |  |  |
| Não disponível | 37 | 17,0 | 1 | 1,2 |  |  |
| **Material para ventilação neonatal** |  |  |  |  |  |  |
| Disponível | 208 | 95.6 | 47 | 98.0 |  |  |
| Não disponível | 10 | 4.4 | 1 | 2.0 |  |  |
| **Unidade transfusional ou Banco de sangue** |  |  |  |  |  |  |
| Sim | 158 | 72.3 | 32 | 66.8 |  |  |
| Não | 60 | 27.7 | 16 | 33.2 |  |  |
| **Testes laboratoriais** |  |  |  |  |  |  |
| Disponível | 191 | 87.8 | 41 | 86.6 |  |  |
| Não disponível | 27 | 12.2 | 7 | 13.4 |  |  |
|  |  |  |  |  |  |  |
| * Hospitais mistos representam 45.6% de todas as instituições e 88.3% dos partos nesses hospitais tem financiamento público | | | | |  |  |
