# Supplementary material for: Use of Robson classification to assess cesarean section rate in Brazil: the role of source of payment for childbirth
Source: Reprod Health. 2016 Oct 17;13(Suppl 3):128. doi: 10.1186/s12978-016-0228-7 (PMC5073850; doi:10.1186/s12978-016-0228-7)
Supplement: Additional file 3: — Reviewer reports. (PDF 293 kb) [file 12978_2016_228_MOESM3_ESM.pdf]

Referee's comments to the authors– this sheet WILL be seen by the author(s) and published with the article

Title	Use of Robson classification to assess cesarean section rate in Brazil: the role of source of payment for childbirth
Author(s)	Marcos Nakamura-Pereira, Maria do Carmo Leal, Ana Paula Esteves Pereira, Rosa Maria Domingues, Jacqueline Alves Torres, Marcos Augusto Dias
Referee's name	Joshua Vogel

When assessing the work, please consider the following points, where applicable:

1. Is the question posed by the authors new and well defined?
2. Are the methods appropriate and well described, and are sufficient details provided to replicate the work?
3. Are the data sound and well controlled?
4. Does the manuscript adhere to the relevant standards for reporting and data deposition?
5. Are the discussion and conclusions well balanced and adequately supported by the data?
6. Do the title and abstract accurately convey what has been found?
7. Is the writing acceptable?

Please make your report as constructive and detailed as possible in your comments so that authors have the opportunity to overcome any serious deficiencies that you find and please also divide your comments into the following categories:

- Major Compulsory Revisions (which the author must respond to before a decision on publication can be reached)
- Minor Essential Revisions (such as missing labels on figures, or the wrong use of a term, which the author can be trusted to correct)
- Discretionary Revisions (which are recommendations for improvement but which the author can choose to ignore)

Where possible please supply references to substantiate your comments.

When referring to the manuscript please provide specific page and paragraph citations where appropriate.

General comments:
Thanks for the opportunity to review. This is a great paper, the use of Robson classification in this national sample is illuminating. Please see some methodological queries below that need to be addressed.

Major compulsory revisions:

- Could you please provide (if available) descriptive characteristics of facilities, stratified by payment type (public/private).
- I note that a significant number of comparisons (chi-square tests) are being made in Table 3. I am not a statistician but the risk of Type 1 error is likely much higher as a result. An adjustment to the test, or lowering of the significance level may be in order.
- I am unclear on how the authors have handled the issue of missingness in the Robson classification. They report that overall missing rate for Robson (group X) was 0.03%. However the denominator for Prev CS in Table 1 suggests a high rate of missing (50%?) for the prev CS variable. Given this is one of the key variables for Robson, how was this handled in the analysis? I assume that women with missing Prev CS were considered to have zero previous CS, if this is the case it should be clearly stated and also acknowledged in limitations (i.e. that it is possible that some women in that group did have a previous CS). It would be good to state why the Prev CS missing rate was so high.
- Table 1 and Table 4 – I am unable to assess if calculations are correct, as for each row only % is provided. Kindly provide n/N (%) for each row.

Minor essential revisions:

- “This study included a complex sample of 266 hospitals” – not sure what you mean by complex in this sentence?
- Methods: While the Birth in Brazil methods are reported elsewhere, please consider some additional clarifying details regarding selection of the facilities and their generalizability, i.e. Were the hospitals representative of all hospitals in the country, and/or were women included representative of all women delivering in hospitals in Brazil?
- it would be good to clarify what aspect of the Births in Brazil data collection methods (medical records, interview etc) were used when obtaining information relating to the Robson classification variables – were all variables from tge interview or only from medical records?
- “The gestational age was calculated using an algorithm that used majority early ultrasound estimates [22].” Could you please rephrase this statement, it is not quite clear what you mean – do you mean that the majority of women had a GA from an algorithm that used both US and LMP, or that the majority of women had an early ultrasound? If you have an estimate of the proportion of women with US-based GA, that would be helpful.
- “We took into consideration the complex sampling design in all statistical analyses.” – could you please provide additional detail on what procedures were used to do this. Also, if clustering is a factor, an adjustment to the significance test (chi-square testing) may also be warranted?
- Ethical considerations – I assume the Births in Brazil study was reviewed by an IRB, but details are not provided.
- In the Results I note the authors tend not to quote specific data values (n, %) with p-values. I think this would be helpful to the reader.
- Results: “Within group 8 and in groups with a 100% CS rate (2b, 4b and 9), there were no differences according to payment type.” This sentence I think doesn’t add a lot of information, as we would expect this to be the case given the CS rates in 2b and 4b are 100%. It could be removed.
- Discussion, main findings: I was struck by the fact that CS rates in pretty much all Robson groups were equal or higher (significantly or non-significantly) in the private sector compared to public sector. Hence the relative overuse of CS in private facilities is not limited to any one situation or population, but is being more liberally used in all women. I think this could be a little more clearly emphasized in the Discussion
- “To our knowledge, it is the third study that used the Robson classification to assess CS rates at a national level and the second to use primary data.” – please provide references for those two other studies.
- “Our results showed that women with private health insurance were more frequently…” – should this sentence refer to “women who delivered in a private facility” – as the private group could include women who paid privately rather than had private insurance, I believe?
- “Our data are consistent with the WHO Global Survey of Latin America, where group 5 accounted for 26.7% of CS.” Please provide reference for this sentence.
- Table 1 – please revise “p value” to “ chi square p value” (or relevant test used)
- Table 2- I think it would be helpful to readers if you explain in the methods or in a footnote the meaning/definitions of relative size, absolute and relative contribution to CS rates. Those not familiar with Robson classification may find it a bit hard to understand.
- The figures on final page needs labels for X and Y axes, and explanation of LRW and HRW
- in some places, “.” Is used and in others “,” is used. Suggest consistency throughout

Discretionary revisions:

- While the first sentence of the introduction makes reference to the 1985 WHO statement, I would ask authors to consider referring to the latest WHO statement on CS rates from 2015, as this has superseded the 1985 statement.
- The paper would benefit from copyediting in a few places.
- I found the Discussion relevant but very long, it became a bit hard for the reader to get through. I would suggest the authors consider how the Discussion could be reduced and more focused on the key messages.

Referee's comments to the authors– this sheet WILL be seen by the author(s) and published with the article

Title	Use of Robson classification to assess cesarean section rate in Brazil: the role of source of payment for childbirth
Author(s)	Marcos Nakamura-Pereira, Maria do Carmo Leal, Ana Paula Esteves Pereira, Rosa Maria Domingues, Jacqueline Alves Torres, Marcos Augusto Dias
Referee's name	Maria Regina Torloni

When assessing the work, please consider the following points, where applicable:

1. Is the question posed by the authors new and well defined?
2. Are the methods appropriate and well described, and are sufficient details provided to replicate the work?
3. Are the data sound and well controlled?
4. Does the manuscript adhere to the relevant standards for reporting and data deposition?
5. Are the discussion and conclusions well balanced and adequately supported by the data?
6. Do the title and abstract accurately convey what has been found?
7. Is the writing acceptable?

Please make your report as constructive and detailed as possible in your comments so that authors have the opportunity to overcome any serious deficiencies that you find and please also divide your comments into the following categories:

- Major Compulsory Revisions (which the author must respond to before a decision on publication can be reached)
- Minor Essential Revisions (such as missing labels on figures, or the wrong use of a term, which the author can be trusted to correct)
- Discretionary Revisions (which are recommendations for improvement but which the author can choose to ignore)

Where possible please supply references to substantiate your comments.

When referring to the manuscript please provide specific page and paragraph citations where appropriate.

General comments: This is an interesting and relevant study using a large dataset and I compliment the authors for their work. Overall, the manuscript is well written and very detailed. I have minor suggestions to improve the clarity and reporting quality of the manuscript.

Minor essential revisions:
Use "labor" (American spelling) or "labour" (British spelling) throughout the text.
Abstract. The objective of the study is clear but the 2nd statement in the same sentence ("...and provide information to guide public policies aimed at reducing CS rates") should be in a separate sentence since this is not an Objective per se but informs about the relevance of the study (i.e. it is an 'implication for practice' statement).
Background
1. Use the more recent (2015) WHO statement on CS rate at population level (current ref 16), instead of the 1985 statement.
2. In the last paragraph of this section, add a sentence specifying clearly what are the Objectives of this study: to "assess and compare differences in CS rates according to source of payment, using the Robson classification".
Methods
3. Add information on the representativeness of the 266 hospitals participating in the Birth in Brazil study, in relation to the whole country. Provide a brief summary of how these hospitals were selected.

Methods, continued
4. Clarify the meaning of “labor (induced and spontaneous)” (yes/no). How can labor be both induced AND spontaneous?
5. How was the information about pre-labor CS collected? How did you construct Robson Groups 2b and 4b? Was it by deduction: for example if a nullipara did not have labor induced (2a) and did not have spontaneous onset of labor (1), then you deduced that she had a pre-labor CS (2b)? Or did you use another method? Please clarify.
6. Define “severe infection during pregnancy”.
7. Under Ethical Considerations, it is unclear whether the authors sought and obtained approval from the institutional review boards of each of the 266 participating hospitals or not. Please clarify this and consider excluding unnecessary details such as the national resolution number and specific protocol number.

Results
8. Specify what were the missing variables in the 7 cases that could not be classified.
Table 1:
-provide the exact numbers before each of the %
-why was education categorized in this way? It does not follow usual national or international categories.
-what is the meaning of “Labor” Yes, No (line after Induction of Labour)? Do you mean Spontaneous onset of labor?
Table 2:
-Align the numbers in the 1st column so that they correspond to the definition in the 2nd column
-In column 2, Robson group 4: the last words should be “CS before labor” and not “CS labor”
-Use “nullipara” and “multipara” or “nulliparous women” and “multiparous women”.

9. 2nd paragraph of Results, this sentence is confusing: “Using the subcategories of group 2, 70% of women delivered by a CS before labor.” You mean that “Group 2 was the single largest group in the study, comprising 20% of the whole population. Within this subset of nulliparas at term with a single cephalic infant, approximately 70% of them were submitted to pre-labor CS and nearly 30% had labor induced.”?

10. I suggest that you modify this sentence “Almost 65% of all CSs performed in Brazil were from groups 2 and 5” to “Almost 65% of the all CS performed in the study population (or sample) were from groups 2 and 5”. This is a sample of the country´s population; it is not representative of the entire country, as you state yourself in the 2nd paragraph of Strengths and Limitations. Throughout the text of Results and Discussion, I recommend that you modify similar statements where the word “Brazil” is used and change this to “sample” or “study population” or “participants”.

11. An additional Limitation of the study, is that it reports data collected in 2011-12 and may not represent current practice. This should be added to the Strengths and Limitations paragraph.

12. Another limitation of the study is the potential misclassification of some women who belonged in Groups 1 and 3 and were erroneously classified as Groups 2 and 4 because of the definition used for labor induction. For instance, it is possible that some nulliparous and multiparous women admitted with spontaneous onset of labor (Groups 1 and 3) received oxytocin before reaching 4 cm dilation, for augmentation of labor. According to the authors´ definition of “induction” (all with < 4 cm dilation using any agent, including oxytocin), these women will have been misclassified as 2a and 4a (instead of 1 and 3, respectively). This could spuriously increase the sizes of groups 2a and 4a and decrease the sizes of groups 1 and 3. Although this probably will not affect the main findings of the study, it should be mentioned.

13. The title of Table 4 is confusing. I suggest that you change it to “Caesarean section rates per Robson group in high and low risk women according to source of payment” or something similar. I think that Figure 1 is not necessary; it simply repeats the same information provided on Table 4.

14. The Interpretation section is very detailed and interesting but too long (11 paragraphs, now). I think it would benefit if it were more concise and written in a more succinct style. The authors could, for example, consider deleting the paragraphs about the trends in CS in Brazil (paragraphs 2 and 10) and also the paragraph about the goups that less contributed to the overall CS rate (paragraph 8).

15. This sentence is confusing and should be rewritten “The two large groups in the public sector (groups 3 and 1) had little importance in the private sector.” You are referring to the relative sizes of the groups in the different sectors?

16, The last paragraph of Conclusion should be toned down and rewritten since it makes some inferences that are not directly supported by the findings of this study. For instance, there is no evidence from this survey that all CS in the private sector are “not indicated for medical reasons”.
